# Blood-invigorating Chinese herbal medicines plus conventional therapy for endometriosis: a network meta-analysis of 107 randomized controlled trials

**DOI:** 10.3389/fmed.2026.1776698

**Published:** 2026-05-13

**Authors:** Weiru Liu, Like Xu, Jing Jia, Min Xu, Yingzhou Tian

**Affiliations:** 1 Second Clinical Medical College, Guangzhou University of Chinese Medicine, Guangzhou, Guangzhou, China; 2 Science and Technology Innovation Center, Guangzhou University of Chinese Medicine, Guangzhou, China

**Keywords:** Chinese botanical medicine, compound Chinese medicine, endometriosis, network meta-analysis, Traditional Chinese medicine

## Abstract

**Objective:**

To systematically evaluate the efficacy and safety of blood-invigorating and stasis-resolving Chinese herbal medicines (BISMs) when combined with conventional clinical therapy for endometriosis, and to provide evidence-based guidance for clinical decision-making.

**Methods:**

Eight databases (CNKI, WanFang, VIP, SinoMed, PubMed, EMBASE, Cochrane Library, and Web of Science) were searched from inception to 16 June 2025 for randomized controlled trials (RCTs). Two reviewers independently screened studies, extracted data, and assessed risk of bias using a modified Cochrane tool. A random-effects network meta-analysis under a frequentist framework was performed with Stata 17.0 (mvmeta command). Surface under the cumulative ranking curve (SUCRA) values were used to rank interventions. Primary outcomes were overall clinical efficacy, pain visual analog scale (VAS), sex hormones (E2, FSH, LH), serum CA125, endometriotic cyst diameter, recurrence rate, and incidence of adverse events.

**Results:**

A total of 107 RCTs were included. 1. Global efficacy: BISMs plus modern medical therapy significantly improved overall response; the combination of Guizhi Fuling pills/capsules with biomedicine ranked first (SUCRA = 87.4%). 2. Symptom relief: Regimens such as Sanjie Zhentong capsules and Xiaojin pills provided an additional 1–2-point reduction in VAS compared with modern medical therapy alone. 3. Hormonal regulation: Bushen Huoxue Sanyu decoction, Dan’e Fukang paste, and Gongliuxiao capsules showed prominent reductions in E2, FSH, and/or LH.4. Inflammation and lesion control: Danggui Shaoyao powder and Shaofu Zhuyu granules ranked highest for lowering CA125 and shrinking cyst diameter. 5. Recurrence and safety: Xiaojin pills, Bushen Huoxue Sanyu decoction, and Shaofu Zhuyu granules markedly reduced recurrence, while Dan’e Fukang paste, Guizhi Fuling pills/capsules, and Sanjie Zhentong capsules were associated with fewer adverse events. Funnel plots revealed no evident publication bias.

**Conclusion:**

Combining BISMs with conventional modern medical treatment offers superior multidimensional benefits—pain alleviation, hormonal balance, inflammatory control, cyst reduction, and recurrence prevention—over modern medical therapy alone, with a favorable safety profile. Among the evaluated regimens, Guizhi Fulingpills/capsules, Dan’e Fukang paste, Shaofu Zhuyu granules, and Gongliuxiao capsules demonstrated the most consistent overall performance. High-quality, large-scale RCTs with long-term follow-up are warranted to confirm and refine these findings.

## 1 Introduction

Endometriosis (EMs) is an estrogen-dependent chronic gynecological disease characterized by the ectopic implantation of active endometrial-like tissue outside the uterine cavity. The periodic bleeding, chronic inflammatory responses, and fibrotic adhesions caused by these ectopic lesions can lead to a series of clinical symptoms. Patients often present with progressive dysmenorrhea, infertility, dyspareunia, and other symptoms (1). Global epidemiological studies have shown that EMs affect approximately 6–13% of women of reproductive age, with a total number of nearly 247 million patients worldwide (2). This disease significantly reduces the quality of life and imposes a heavy socioeconomic burden: relevant research data from Europe and the United States indicate that the direct medical cost per patient is approximately 1,459–20,239 US dollars per person per year (3).

Currently, a large number of studies (4–6) recommend hormonal suppression therapy (including oral contraceptives, progestogens, dienogest, etc.) or surgical resection as first-line treatment options. However, hormonal therapy is often accompanied by adverse reactions such as menstrual abnormalities, weight gain, and emotional depression (7, 8), and the problem of high recurrence rate after drug withdrawal has not been fundamentally solved. Although surgical treatment can quickly relieve pain, it also carries risks such as damage to ovarian reserve function and recurrence of postoperative adhesions (9). Therefore, there is an urgent clinical need to develop new therapeutic strategies that are safer, more effective, and suitable for long-term application, as alternatives or supplements to existing therapies.

In the system of traditional Chinese medicine (TCM) theory, endometriosis (EMs) falls into the categories of diseases such as “dysmenorrhea,” “abdominal mass (zhengjia),” and “infertility”—classic TCM disease terms describing pelvic pain, abnormal mass formation, and reproductive dysfunction, respectively. Its core pathogenesis is “internal obstruction by blood stasis,” meaning that the blood that has escaped from the meridians stagnates in the uterus and uterine collaterals, forming pathological masses (zhengji), blocking the circulation of qi (vital energy) and blood, and leading to pain and infertility. A classic TCM text *Zhu Bing Yuan Hou Lun* (Treatise on the Causes and Manifestations of Various Diseases) records: “Blood stasis inside the body. if the stasis remains unresolved for a long time, it will turn into accumulations and masses,” which is highly consistent with the pathological features of pelvic adhesions and cyst formation in EMs patients. In recent years, TCM’s understanding of the pathogenesis of EMs has been continuously deepened, evolving from the single theory of “blood stasis” to complex pathogenesis theories such as “kidney deficiency with blood stasis (a combination of constitutional weakness and pathological stasis),” “intermingled phlegm and blood stasis (mucus-like pathological substances combined with stasis),” and “accumulation of stasis and toxin (long-term stasis leading to pathogenic toxin formation).”

The core therapeutic approach for EMs in TCM is promoting blood circulation and removing blood stasis. This approach targets the root pathological change of stasis to restore normal qi and blood circulation, and can improve the disease state through multiple pathways, including promoting microcirculation, inhibiting the proliferation of ectopic endometrium, regulating inflammatory and immune imbalances, and reducing prostaglandin levels. Basic research has shown that active ingredients of blood-activating and stasis-removing Chinese herbs, such as tanshinone IIA from *Salvia miltiorrhiza*, can significantly reduce serum levels of TNF-α and IL-1β, downregulate the expression of ICAM-1, MMP-9, and VEGF in ectopic uterine tissues, reduce lesion formation, and exert therapeutic effects by inhibiting the activation of the PI3K/Akt/mTOR signaling pathway (10).

Previous network meta-analyses have mostly regarded oral commercial Chinese polyherbal preparations (CCPP) of the blood-activating and stasis-removing category as a single intervention group, integrating data from dozens of randomized controlled trials to compare and rank the efficacy of commonly used CCPPs. However, such analyses mainly focus on comparing commercially available CCPPs combined with conventional treatment versus conventional treatment alone. There is still a lack of comprehensive network meta-analyses that cover both CCPPs and classic compound prescriptions, resulting in unclear relative efficacy of various traditional Chinese medicine therapeutic strategies in EMs. This limits the evidence support for clinical decision-making in terms of treatment selection and optimal combination.

This study intends to systematically evaluate the efficacy of blood-activating and stasis-removing drugs in the treatment of EMs through a network meta-analysis and compare them with other common treatment methods. The aim is to provide a scientific evidence-based basis for clinical practice, not only to assess the objective efficacy of blood-activating and stasis-removing drugs, but also to explore their relative advantages among various traditional Chinese medicine regimens. By integrating data from multiple studies, it will further guide the optimization of clinical individualized treatment strategies and provide theoretical support for the design of subsequent clinical trials.

Blood-invigorating effects are assessed through multiple primary outcome parameters that reflect improvements in blood circulation, reduction of stasis, and alleviation of related symptoms: 1. Clinical efficacy: Overall effective rate based on symptom improvement (dysmenorrhea relief, reduced pelvic pain) and lesion regression. 2. Pain relief: VAS score reduction, directly reflecting the alleviation of pain caused by blood stasis obstruction. 3. Inflammatory and lesion markers: Reduction in serum CA125 levels, an indicator of endometriosis-related inflammation and lesion activity. 4. Lesion regression: Shrinkage of endometriotic cyst diameter, indicating absorption of stasis-related lesions. 5. Hormonal balance: Regulation of E2, FSH, and LH levels, as hormonal imbalances are closely linked to blood stasis and ectopic tissue proliferation. 6. Recurrence rate: Long-term prevention of disease recurrence, reflecting the sustained improvement of blood circulation and resolution of stasis.

## 2 Materials and methods

### 2.1 Literature search strategy

Computer-based searches will be conducted in both Chinese and English databases, including China National Knowledge Infrastructure (CNKI), WanFang Data Knowledge Service Platform (WanFang), VIP Chinese Science and Technology Periodical Database (VIP), SinoMed (Chinese Biomedical Literature Database), as well as PubMed, EMBASE, Cochrane Library, and Web of Science. The search timeframe will cover from the establishment of each database to June 16, 2025. According to the characteristics of different databases, searches will be performed using a combination of subject terms and free words.

Chinese search terms mainly include “zigongneimoyiweizheng” (endometriosis), “neiyizheng” (endometriosis, abbreviated), “zigongneimoyiweinangzhong” (endometriotic cyst), “qiaokelinangzhong” (chocolate cyst), “zhongcaoyao” (Chinese herbal medicine), “zhongchengyao” (Chinese patent medicine), “zhongyaotangji” (Chinese herbal decoction), “suijiduizhaoshiyan” (randomized controlled trial), “suijifenpei” (random allocation), “linchuangshiyan” (clinical trial), etc.

English search terms include “endometriosis,” “chocolate cyst,” “Chinese patent medicine,” “Chinese herbal decoction,” “randomized controlled trial (RCT),” “RCT,” etc.

Meanwhile, the reference lists of included literatures will be manually searched to ensure the comprehensiveness of the literature retrieval. To further guarantee the thoroughness of the search, supplementary manual searches will also be conducted on the reference catalogs of the included literatures.

### 2.2 Inclusion criteria

The included literatures must meet the following criteria simultaneously:

#### 2.2.1 Study type

Limited to Randomized Controlled Trials (RCTs), without restrictions on the type of blinding or multi-center settings.

#### 2.2.2 Study subjects

Participants must be patients with a clear diagnosis of endometriosis, with no restrictions on diagnostic criteria, and no limitations on basic characteristics such as age and disease duration.

#### 2.2.3 Intervention measures

The experimental group receives blood-activating and stasis-removing Chinese medicines on the basis of conventional clinical drug treatment, including oral commercial Chinese polyherb preparations or Chinese herbal compound decoctions. No other non-pharmacological TCM interventions are combined during the treatment process.

#### 2.2.4 Control measures

The control group receives conventional clinical drug medical treatment (such as hormonal drug therapy, etc.) without interventions of TCM.

#### 2.2.5 Limitations on the scope of Chinese medicines

The Chinese herbal compounds or commercial preparations used in the included studies must have a clear effect of “activating blood circulation and removing blood stasis,” which is defined according to the records in the Pharmacopoeia or the descriptions of the original authors.

#### 2.2.6 Primary outcome indicators

The literature must report at least one of the following primary outcome indicators, including: total clinical effective rate; sex hormone indicators [such as follicle-stimulating hormone (FSH), luteinizing hormone (LH), estradiol (E2), etc.]; serum CA125 level; diameter of endometriotic cyst; recurrence rate after treatment; incidence of adverse reactions.

### 2.3 Exclusion criteria

Studies of the following types or those failing to meet the criteria will be excluded:

#### 2.3.1 Non-RCT studies

Including observational studies (such as cohort studies, case-control studies, etc.), case reports, review articles, animal experiments, and other non-clinical studies.

#### 2.3.2 Chinese medicine interventions not meeting requirements

Literatures where the Chinese medicine preparations used in the study do not have the effect of activating blood circulation and removing blood stasis, or where their efficacy attribution cannot be determined.

#### 2.3.3 Incomplete outcome indicators

Lack of reports on the above-mentioned primary outcome indicators, or incomplete relevant data that cannot be supplemented by contacting the original authors.

#### 2.3.4 Combination with other intervention methods

Those involving non-pharmacological TCM treatments such as acupuncture, tuina (Chinese massage), and application therapy during the intervention process.

#### 2.3.5 Duplicate data

For literatures with duplicate publications or overlapping data sources, if multiple studies have overlapping content, the one with a later publication date or higher research quality will be included.

#### 2.3.6 Unclear preparation composition

Studies that do not report the detailed composition of the Chinese herbal preparations or commercial Chinese polyherbal preparations used.

### 2.4 Data extraction and quality assessment

Two researchers will independently screen the literature and extract data in accordance with the inclusion and exclusion criteria, then cross-check the results. Discrepancies will be resolved through discussion or arbitration by a third party. The extracted content includes basic study information (authors, publication year, etc.), characteristics of participants, sample size, specific protocols of intervention and control measures (including names of Chinese medicines, detailed composition, and treatment courses, and drug interactions etc.), primary outcome indicators and their result data, adverse reactions, etc. Meanwhile, key methodological information that may affect the risk of bias will be recorded. Subsequently, the modified Cochrane Risk of Bias tool will be used to evaluate the quality of included studies, covering aspects such as random sequence generation, allocation concealment, blinding (blinding of participants, personnel, and outcome assessors), completeness of outcome data, and selective reporting. Each assessment result will be classified into “low risk of bias,” “unclear risk,” or “high risk of bias.” The quality assessment will be independently completed by two evaluators, and any disagreements will be resolved by a third party.

### 2.5 Taxonomic validation of plant species

All plant species used in the included preparations were taxonomically validated using the Medicinal Plant Names Services (MPNS)^1^ and Plants of the World Online.^2^ The full species name, including authorities and family, as well as the pharmacopeial drug name (if assigned) were recorded, following the format: [Scientific name with authority] [Family; Pharmacopeial drug name].

### 2.6 Subgroup analysis based on preparation composition

Subgroup analysis was conducted based on the composition of the preparations. Preparations were grouped according to their core botanical drugs and therapeutic mechanisms (e.g., preparations containing Salvia miltiorrhiza, preparations with kidney-tonifying and blood-stasis-resolving effects). Efficacy and safety outcomes were compared among subgroups to explore the impact of different compositions on treatment effects.

### 2.7 Statistical methods

Stata 17.0 software will be used for network meta-analysis, and the mvmeta command will be applied to perform multivariate network meta-analysis under the frequentist framework. For continuous outcome indicators, the mean difference (MD) will be used for statistical analysis; for binary outcome indicators, the risk ratio (RR) will be used for evaluation. A random-effects model will be adopted, with between-study heterogeneity estimated by the restricted maximum likelihood (REML) method, and the pooled effect size and its 95% confidence interval (CI) will be calculated. Heterogeneity will be represented by τ^2^ and classified according to the following criteria: low heterogeneity (< 0.04), low to moderate heterogeneity (0.04–0.16), moderate to high heterogeneity (0.16–0.36), and high heterogeneity (> 0.36). Stata software will be used to draw network plots, and the surface under the cumulative ranking curve (SUCRA) will be calculated to rank and compare the efficacy of interventions. Funnel plots will be used to determine the presence of potential small-sample effects or publication bias.

A random-effects model will be used for comprehensive data analysis. The effect size indicators will be selected according to the type of outcome indicators: relative risk (RR) or odds ratio (OR) will be used for binary outcomes, and mean difference (MD) or standardized mean difference (SMD) will be used for continuous outcomes. The 95% confidence interval (CI) or Bayesian 95% credible interval (CrI) for each effect size will be calculated. The surface under the cumulative ranking curve (SUCRA) values will be used to rank the efficacy of each intervention scheme and evaluate the relative effectiveness of different treatments on each primary outcome. If necessary, this study will also conduct potential publication bias tests to ensure the robustness and reliability of the results.

## 3 Results

### 3.1 Literature search process and results

**FIGURE 1:**
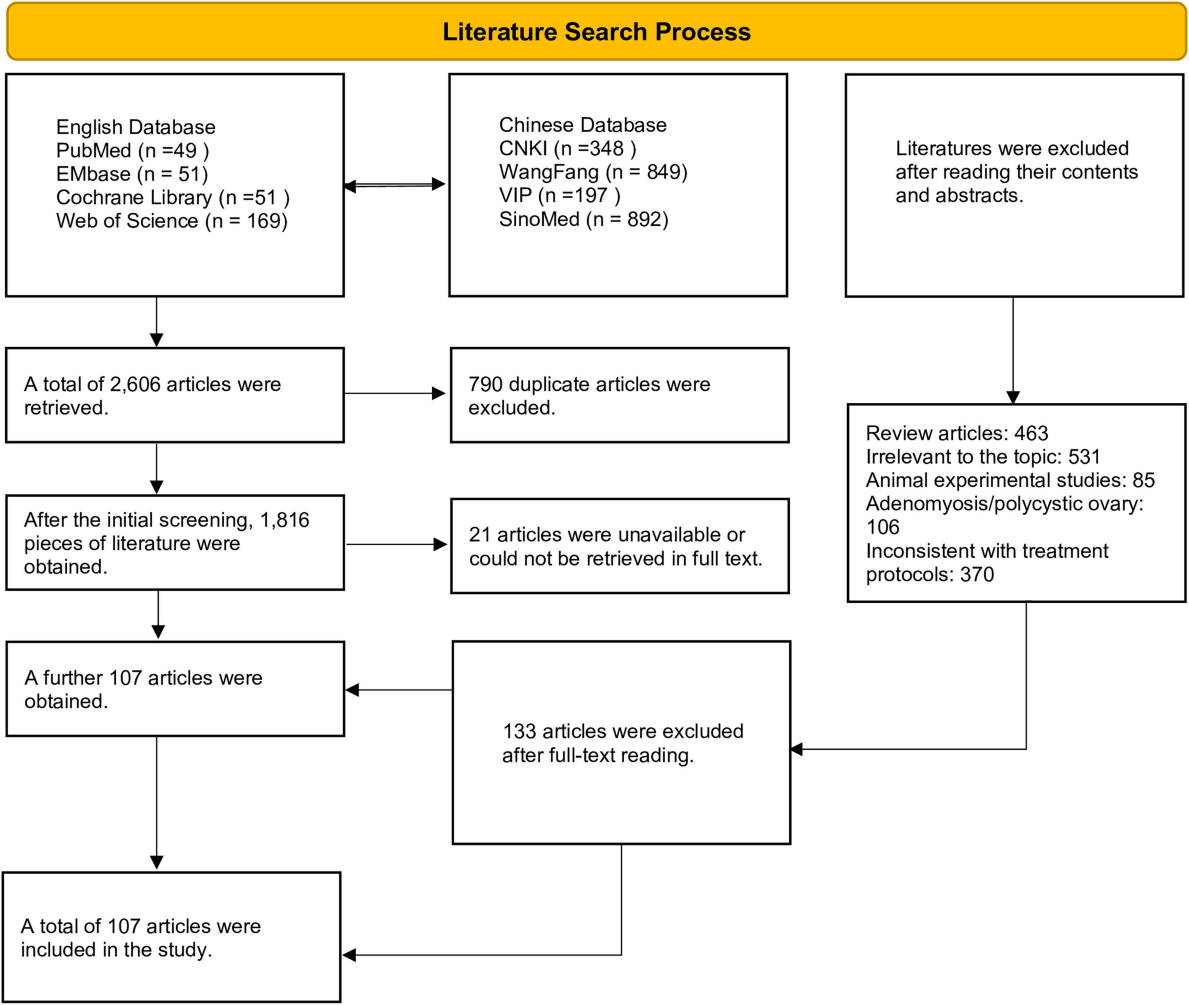
Flow chart of literature retrieval.

A total of 2,606 articles were retrieved through database searches. All articles were imported into Endnote X9 for sorting, with 790 duplicate articles excluded, leaving 1,816 articles. The titles and abstracts of the remaining articles were rapidly screened, and 1,555 articles irrelevant to the research topic or failing to meet the initial screening criteria were excluded. After the initial screening, 240 articles were retained for secondary screening. Further screening through full-text reading excluded 133 articles that did not meet the inclusion criteria, and finally 107 articles were included for this network meta-analysis. The literature retrieval and screening process underlying this study is detailed in Figure 1.

### 3.2 Basic characteristics of included literatures

A total of 107 literatures included in this research were all two-arm studies, with the total sample sizes of the experimental group and the control group being 5,256 cases and 4,873 cases, respectively. The ages of patients were concentrated at 32.01 ± 3.71 years (experimental group) and 31.89 ± 4.20 years (control group), with an average disease duration of 3.28 ± 1.10 years (experimental group) and 4.31 ± 1.47 years (control group); some studies did not provide data on age and disease duration. In terms of intervention measures, a total of 22 interventions were involved, including Chinese patent medicines or compound prescriptions combined with conventional medical treatment, with treatment cycles ranging from 3 to 7 months. Besides, among these 107 researches, 10 employed Chinese herbal medicines or integrated Chinese and conventional medicine for the prevention of recurrence after EMs surgery (11–20), while all other studies involved non-surgical patients who received pharmacological conservative treatment alone. The basic characteristics of the literatures are detailed in Table 1.

**TABLE 1:** Basic characteristics of included literatures.

Included articles	Sample size	Average age (years)	Duration of disease (years)	Course of treatment (months)	Interventions		Outcome indicators
	(T/C)	(T/C)	(T/C)		T	C	
(21)	46/46	34.52 ± 4.28/33.92 ± 4.07	1.90 ± 0.48/1.97 ± 0.53	3	N + V	V	➀➁➄➆
(22)	33/33	33.75 ± 5.62/33.75 ± 5.62	1.35 ± 1.74/1.35 ± 1.74	3	F	V	➂➅
(23)	53/52	33.51 ± 3.24/33.53 ± 3.22	2.25 ± 0.67/2.23 ± 0.68	3	N + V	V	➀➂➆
(24)	61/60	32.08 ± 5.37/31.86 ± 5.23	/	3	N + V	V	➀➁➂➄➆
(25)	31/31	/	/	3	O	V	➀➁
(26)	31/31	38.50 ± 6.41/38.00 ± 6.33	/	3	D + V	V	➀➁
(27)	25/25	20∼40/20∼40	0.42 ± 1.17/0.42 ± 1.17	3	A	V	➀
(28)	45/45	32.1 ± 3.9/32.8 ± 4.3	/	3	R + V	V	➀➁➂➃➄➆
(29)	36/36	36.17 ± 3.35/35.39 ± 4.43	4.19 ± 1.49/3.37 ± 1.23	3	H + V	V	➀➂
(30)	45/45	36.10 ± 3.09/36.02 ± 3.12	1.54 ± 0.29/1.48 ± 0.26	3	B + V	V	➀➁➄➆
(31)	46/46	35.65 ± 4.58/36.05 ± 4.69	2.62 ± 0.73/2.69 ± 0.75	3	U + V	V	➁
(32)	99/99	31.3 ± 8.2/33.4 ± 8.0	2.4 ± 1.2/2.8 ± 1.4	6	H + V	V	➀➂
(33)	42/28	34.5/32.5	5.5/5.2	3	Q	V	➀
(34)	43/43	27.93 ± 6.08/27.83 ± 6.11	2.71 ± 0.62/2.65 ± 0.69	3	N + V	V	➀➁➂➅
(35)	47/47	34.2 ± 8.2/35.1 ± 8.6	/	3	Q	V	➀
(36)	30/30	30 ± 8/31 ± 6	4.5 ± 2.0/4.4 ± 1.8	3	H + V	V	➀➂
(37)	39/39	36.5/36.5	/	3	H + V	V	➀➂
(38)	45/45	30.9 ± 5.5/30.2 ± 6.1	2.8 ± 0.4/3.0 ± 0.8	6	C + V	V	➀➁➂➃➅➆
(39)	38/38	37.4 ± 4.5/34.5 ± 5.1	3.3 ± 0.6/3.6 ± 0.2	3	C + V	V	➀➅➆
(40)	30/30	30/30	6/6	3	K	V	➀➆
(41)	25/25	31.88 ± 5.63/29.68 ± 4.46	1.52 ± 1.74/15.36 ± 18.63	3	F	V	➃➄
(42)	26/28	34.85 ± 6.09/36.89 ± 4.95	6.12 ± 2.53/6.36 ± 2.47	3	E	V	➁➃➆
(43)	45/45	32.46 ± 3.24/32.13 ± 3.17	3.18 ± 1.02/3.24 ± 1.05	3	H + V	V	➀➂
(44)	30/30	22∼45/21∼47	1∼25/1∼26	3	F	V	➁
(45)	34/33	31.5 ± 7.8/30.9 ± 8.2	5.5 ± 6.4/5.7 ± 6.8	3	B	V	➁
(18)	45/45	35.28 ± 5.62/36.08 ± 5.34	4.53 ± 1.22/5.08 ± 1.06	3	A	V	➃
(46)	27/27	28.7 ± 5.0/29.9 ± 4.2	2.43 ± 0.69/2.65 ± 0.71	3	Q + V	V	➀➁➆
(47)	45/45	31.90 ± 4.13/31.34 ± 4.29	1.53 ± 0.33/18.10 ± 3.71	6	R + V	V	➀➂➆
(48)	34/34	33.08 ± 11.35/34.3 ± 12.23	10.84 ± 3.61/9.7 ± 2.69	/	M + V	V	➀➁
(49)	63/63	30.2 ± 4.1/29.9 ± 4.1	/	3	H + V	V	➀➆
(50)	45/45	18–44/18–44	/	6	C + V	V	➀
(51)	25/25	36.47 ± 6.85/37.38 ± 6.77	3.52 ± 1.10/3.50 ± 1.10	4	H + V	V	➀➁➂➆
(52)	72/58	33.8 ± 7.3/33.8 ± 7.3	/	6	C	V	➀➃
(53)	31/31	28.3 ± 4.6/26.4 ± 3.8	/	3	O	V	➀➁➆
(54)	41/41	34.3 ± 2.0/33.8 ± 2.4	2.1 ± 0.5/2.3 ± 0.7	6	H + V	V	➀➁➂➄➆
(55)	49/49	30.9 ± 3.8/30.3 ± 3.7	3.3 ± 0.9/3.8 ± 1.2	6	N + V	V	➀➁➂➃➄➅
(56)	70/70	33 ± 3.8/33 ± 3.8	/	3	S + V	V	➀➃
(19)	41/41	35.02 ± 2.87/34. 29 ± 2.65	4. 25 ± 1. 14/4. 13 ± 1. 06	3	A + V	V	➀➄
(57)	87/87	30.14 ± 3.98/30.28 ± 4.32	3.76 ± 0.78/3.83 ± 0.84	3	C	V	➀➁➃
(58)	41/41	31.38 ± 7.02/33.02 ± 7.81	1.12 ± 0.37/13.92 ± 4.17	6	C + V	V	➀➂➆
(59)	88/87	29.04 ± 6.31/11.94 ± 3.13d	12.05 ± 2.98/	3	H + V	V	➀➁➂➃➄➆
(60)	54/54	29.25 ± 5.13/29.31 ± 5.24	2.87 ± 0.70/2.93 ± 0.62	3	G + V	V	➀➁➄➆
(61)	50/50	35.58 ± 4.52/36.39 ± 4.12	10.56 ± 1.84/11.21 ± 2.04	6	O + V	V	➀➆
(62)	43/43	29.17 ± 3.28/28.76 ± 3.92	1.02 ± 0.21/0.99 ± 0.22	6	H + V	V	➀➂➃
(63)	40/40	33.86/33.85	/	3	A + V	V	➀➁➃
(64)	100/100	29.8 ± 5.2/28.6 ± 6.9	0.63 ± 0.23/0.62 ± 0.24	6	E	V	➀➁➃
(65)	38/38	34.1/33.6	/	6	L	V	➀➁
(66)	35/35	31.0 ± 3.9/30.9 ± 2.9	3.32 ± 1.18/3.56 ± 1.33	3	O	V	➀➃
(67)	40/40	36 ± 5/37 ± 5	2.6 ± 0.8/2.6 ± 0.9	6	N + V	V	➀➁➃➄➆
(68)	56/49	31.8 ± 5.6/32.9 ± 4.6	/	3	P	V	➀➆
(69)	65/65	34.29 ± 4.48/33.36 ± 4.57	3.59 ± 0.91/3.61 ± 0.82	3	G + V	V	➀➁➂➅➆
(70)	36/36	31.7 ± 6.2/32.3 ± 5.9	/	3	L	V	➀
(71)	23/22	30.5 ± 7.2/31.1 ± 6.7	/	3	H	V	➀➅➆
(72)	116/46	32.6/32.6	4.5/4.5	3	O	V	➀➅➆
(12)	60/47	32.5 ± 6.50/34.6 ± 5.60	/	6	O	V	➀➆
(73)	50/50	32 ± 7/33 ± 5	2.6 ± 1.8/2.7 ± 2.0	3	H + V	V	➀➂
(74)	65/32	33.4 ± 5.8/33.7 ± 6.4	/	6	F	V	➀➄
(75)	34/34	32.55 ± 5.08/33.15 ± 4.98	1.81 ± 0.71/1.93 ± 0.67	3	A + V	V	➀➆
(76)	50/50	28.5 ± 5.2/28.4 ± 5.4	/	3	H + V	V	➀➂
(15)	30/30	20∼50/21∼49	1∼10/1∼9	6	H + V	V	➀➅➆
(77)	87/56	/	/	3	C	V	➀➁
(78)	43/43	32.70 ± 3.23/32.13 ± 3.47	2.93 ± 0.51/2.93 ± 0.51	3	J + V	V	➀➃➆
(17)	81/81	35.2 ± 2.5/36.5 ± 1.7	0.9∼5/0.4∼4.8	6	T + V	V	➀➂➃➅
(13)	62/62	31.2 ± 0.58/30.1 ± 0.78	2.0 ± 0.6/2.2 ± 0.8	6	H + V	V	➀➁➃➄➆
(79)	60/60	31.46 ± 5.84/31.46 ± 5.84	/	3	P	V	➀➂
(80)	30/30	31.9 ± 2.6/32.3 ± 2.7	3.1 ± 0.5/3.2 ± 0.5	3	H + V	V	➆
(81)	58/58	32.15 ± 3.18/33.21 ± 3.68	3.35 ± 0.33/3.56 ± 0.36	6	R + V	V	➀➂➅
(14)	47/47	30.1 ± 3.4/29.8 ± 3.1	1.18 ± 0.19/1.16 ± 0.20	6	A + V	V	➀➂➅
(82)	28/16	/	/	6	O + V	V	➀➆
(83)	40/40	28.15/30	7/6	6	C	V	➀
(84)	30/30	35.3/34.02	5.8/4.9	3	O	V	➀➆
(16)	37/37	34.94 ± 5.70/34.38 ± 5.62	/	6	A + V	V	➀➂➃
(85)	52/38	29.3/30.2	0.50 ± 0.83/0.50 ± 0.75	3	I	V	➀➆
(86)	30/30	33.57 ± 5.99/32.73 ± 5.46	2.60 ± 1.04/2.33 ± 1.03	3	K + V	V	➀➁
(87)	49/49	34.02 ± 2.17/33.41 ± 2.64	5.76 ± 1.21/5.89 ± 1.53	3	M + V	V	➀➃
(88)	52/52	36.92 ± 4.28/36.70 ± 4.36	3.81 ± 0.79/3.69 ± 0.82	3	B + V	V	➀➂➄
(89)	52/52	28.96 ± 4.75/28.85 ± 4.81	2.77 ± 0.53/2.76 ± 0.54	3	D + V	V	➀➁➂➃➄
(90)	39/39	29.98 ± 4.61/29.84 ± 4.85	2.69 ± 0.84/2.71 ± 0.84	3	U + V	V	➀➁
(91)	26/20	25 45/25 45	0.33 ± 0.50/0.33 ± 0.50	3	S	V	➀
(11)	46/40	/	/	6	L	V	➀➅
(92)	146/60	36/36	/	3	C	V	➀
(93)	41/41	32.36 ± 1.39/32.13 ± 1.31	3.82 ± 0.41/3.67 ± 0.28	6	R + V	V	➀➁➂➃
(94)	48/48	35.4 ± 4.5/35.0 ± 3.9	4.3 ± 1.3/4.2 ± 1.1	6	O + V	V	➀➁
(95)	60/60	28.5/30	7/5	6	C	V	➀
(96)	45/45	28.47 ± 5.23/28.03 ± 5.51	5.17 ± 0.82/5.03 ± 0.74	3	H	V	➀➂➃➆
(20)	50/50	34.8 ± 6.4/36.1 ± 6.8	2.6 ± 1.5/2.9 ± 1.8	3	A + V	V	➀➄
(97)	75/75	30.15 ± 3.21/30.25 ± 3.18	3.25 ± 0.69/3.35 ± 3.27	6	C	V	➀➁➆
(98)	75/75	26.11 ± 4.33/26.83 ± 4.19	2.31 ± 0.69/2.19 ± 0.61	6	O + V	V	➀➄➆
(99)	28/28	36.8 ± 4.5/36.2 ± 4.1	1.8 ± 0.7/1.5 ± 0.4	3	H	V	➀➆
(100)	41/41	37.46 ± 6.41/37.35 ± 6.38	4.19 ± 1.17/4.23 ± 1.21	3	J + V	V	➀➃➄➆
(101)	42/42	35.12 ± 1.39/35.89 ± 1.45	3.27 ± 0.92/36.15 ± 1.56	3	G + V	V	➀➂➃
(102)	40/40	33.5/34	7.5/6.5	3	M	V	➀➅
(103)	39/39	27.89 ± 1.27/27.76 ± 1.18	3.78 ± 1.52/3.68 ± 1.35	3	C + V	V	➀➁➂➃
(104)	108/41	34.6/34.6	4.9/4.9	3	C	V	➀➅➆
(105)	68/67	31.08 ± 5.13/30.14 ± 4.56	3.47 ± 2.05/3.24 ± 1.89	6	A + V	V	➀➂➃➅
(106)	30/30	36.5 ± 1.1/36.1 ± 0.9	2.2 ± 0.3/2.1 ± 0.1	3	H + V	V	➁➂➄
(107)	49/49	30.68 ± 15.23/31.15 ± 14.76	5.26 ± 2.07/5.34 ± 2.15	3	I + V	V	➀➂
(108)	60/60	35.25 ± 4.12/35.01 ± 3.39	2.15 ± 0.82/2.27 ± 0.73	6	R + V	V	➀➂➅➆
(109)	40/40	33.05 ± 9.84/32.37 ± 10.65	3.65 ± 2.77/3.41 ± 2.55	3	U + V	V	➀➁
(110)	40/40	36.82 ± 5.03/36.05 ± 4.25	/	3	A	V	➀
(111)	41/41	35.87 ± 3.86/36.98 ± 3.97	/	7	B + V	V	➀➂➃➆
(112)	45/45	29.8 ± 4.2/31.7 ± 7.1	/	6	O + V	V	➀➁➃➆
(113)	45/45	30.35 ± 4.63/30.38 ± 4.65	3.54 ± 0.62/3.56 ± 0.65	6	O + V	V	➀➂➃
(114)	65/62	35.6 ± 4.3/34.5 ± 4.2	3.0 ± 1.0/2.9 ± 0.9	3	G + V	V	➀➁➃➄➆
(115)	60/59	28.3 ± 2.2/29.4 ± 1.9	2.48 ± 0.49/2.37 ± 0.53	6	G + V	V	➀➁➃
(116)	41/39	29.95 ± 3.59/29.39 ± 4.15	3.51 ± 0.72/3.47 ± 0.51	3	G + V	V	➀➁➂➆
(117)	41/41	30.0 ± 3.4/29.8 ± 3.6	4.2 ± 0.8/4.2 ± 0.7	3	G + V	V	➀➁➂

Interventions: A: Bushen Huoxue Sanyu Decoction. B: Dahuang Zhechong Capsule. C: Dan’e Fukang Decoction Extract. D: Dingkun Pill. E: Danggui Shaoyao Powder. F: Eleng Capsule. G: Gongliuxiao (Preparation). H: Guizhi Fuling Pill/Capsule. I: Guizhi Fuling Decoction. J: Hongjin Xiaojie (Preparation). K: Hongteng Formula. L: Jingtong Yushu Granule. M: Shaofu Zhuyu Decoction. N: Shaofu Zhuyu Granule. O: Sanjie Zhentong Capsule. P: Tongjingling Granule. Q: Xuefu Zhuyu Decoction. R: Xiaojin Capsule/Tablet. S: Xiaoyi Decoction. T: Xiaozheng Decoction. U: Xiaozheng Zhitong Decoction. V: Conventional Clinical Drug Therapy. Outcome Indicators: ➀: Total effective rate. ➁: VAS score. ➂: Sex hormone indicators (estradiol [E2], follicle-stimulating hormone [FSH], luteinizing hormone [LH]). ➃: Relevant laboratory serum indicators (e.g., carbohydrate antigen 125 [CA125], etc.). ➄: Diameter of ectopic cyst. ➅: Recurrence rate. ➆: Adverse reactions.

### 3.3 Composition of key preparations

The detailed composition of key preparations such as Dante antigen 125 [CA125], etc.). Fukang Decoction Extract. D: Dingkun Pill. E: Danggui Shaoyao Powder. F: Ele are composed of botanical drugs with well-defined profiles based on pharmacopeial monographs.

Animal medicinal materials (e.g., Moschus, Scolopendra) are labeled with their source families and medicinal parts in accordance with the Chinese Pharmacopoeia (2020 Edition).

The composition of each formula is based on reports from original studies and records in the Chinese Pharmacopoeia, ensuring clarity and consistency with clinical applications.

For formulas with the same name but different dosage forms (e.g., Pill/Capsule, Decoction/Granule), the core components remain consistent, with differences only in excipients or preparation processes. Thus, the core composition is listed uniformly.

### 3.4 Taxonomic validation of plant species

All plant species used in the included preparations were successfully taxonomically validated. The full taxonomic information and pharmacopeial drug names are provided in Supplementary Table 1.

### 3.5 Subgroup analysis results

Subgroup analysis based on preparation composition showed that:

Preparations containing Salvia miltiorrhiza Bunge [Lamiaceae; Salviae miltiorrhizae radix et rhizoma] (e.g., Dan’e Fukang Decoction, Gongliuxiao Preparation) had significantly better effects in reducing CA125 levels and cyst diameter (MD = −1.82, 95%CI: −2.56, −1.08; MD = −0.98, 95%CI: −1.52, −0.44, respectively) compared with preparations without Salvia miltiorrhiza.

Kidney-tonifying and blood-stasis-resolving preparations (e.g., Bushen Huoxue Sanyu Decoction) were more effective in reducing recurrence rate (OR = 0.21, 95%CI: 0.10, 0.44) than preparations focusing solely on blood-stasis-resolving.

Preparations with a combination of Angelica sinensis, Ligusticum chuanxiong, and Paeonia lactiflora showed superior pain relief (VAS score reduction: MD = −1.76, 95%CI: −2.43, −1.09) compared with other composition subgroups.

### 3.6 Quality assessment of included literatures

**FIGURE 2:**
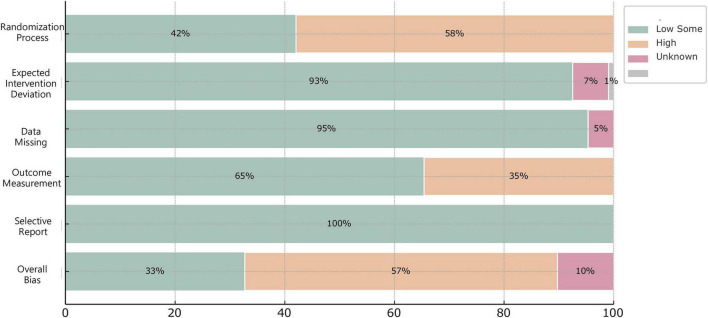
Graph of literature quality bias assessment.

In this study, risk of bias assessment (ROB.2) was conducted for all included randomized controlled trials. Regarding randomization, 62 studies mentioned the term “random” but failed to provide specific randomization methods, thus being assessed as having a moderate risk of bias. In terms of data completeness, 5 studies had sample missingness and did not clearly describe the use of statistical methods such as ITT (Intention-to-Treat) for supplementation or correction, resulting in a high risk of bias. Additionally, all included studies showed no significant risks of bias in data missingness, outcome measurement, or other aspects. Overall, among the 107 studies, 35 were assessed as having a low risk of bias and 61 as having a moderate risk of bias, indicating good methodological quality in terms of randomization procedures, data completeness, outcome reporting, and measurement. However, 11 studies still presented a high risk of bias, mainly reflected in unclear randomization methods, intervention deviations, improper handling and reporting of missing data. The results are detailed in Figure 2 and Table 2.

**TABLE 2:** Quality evaluation table of included articles.

Literature	Randomization process	Intervention deviation	Data missing	Outcome measurement	Selective reporting	Overall bias
Bai and Yan (21)	Low	Low	Low	Low	Low	Low
Cheng and Cao (22)	Some	Low	Low	Low	Low	Some
Chen (23)	Some	Low	Low	Low	Low	Some
Chen (24)	Some	Low	Low	Low	Low	Some
Chen et al. (25)	Some	Low	High	Low	Low	High
Chen et al. (26)	Low	Low	Low	Low	Low	Low
Chen (27)	Low	Low	Low	Low	Low	Low
Li and Li (28)	Some	Low	Low	Low	Low	Some
Ding et al. (29)	Some	Low	Low	Low	Low	Some
Ding (30)	Low	Low	Low	Low	Low	Low
Du (31)	Low	Low	Low	Low	Low	Low
Fan et al. (32)	Low	Low	Low	Low	Low	Low
Fang (33)	Low	High	Low	Low	Low	High
Feng and Guo (34)	Some	Low	Low	Some	Low	Some
Fu and Xie (35)	Low	Low	Low	Some	Low	Some
Gong (36)	Some	Low	Low	Some	Low	Some
Guo (37)	Some	Low	Low	Some	Low	Some
Hu and Kuang (38)	Some	Low	Low	Some	Low	Some
Huang et al. (39)	Low	Low	Low	Some	Low	Some
Huang et al. (40)	Some	Low	Low	Low	Low	Some
Huang et al. (41)	Some	Low	High	Low	Low	High
Huang (42)	Some	Low	Low	Low	Low	Some
Jin et al. (43)	Low	Low	Low	Low	Low	Low
Ju and Jin (44)	Some	Low	Low	Low	Low	Some
Li (45)	Some	Low	Low	Low	Low	Some
Han (18)	Some	Low	Low	Low	Low	Some
Li et al. (46)	Some	Low	Low	Low	Low	Some
Li et al. (47)	Some	Low	Low	Low	Low	Some
Li (48)	Low	Low	Low	Low	Low	Low
Li and Zhang (49)	Low	Low	Low	Low	Low	Low
Li and Zhu (50)	Low	Low	Low	Low	Low	Low
Li (51)	Some	Low	Low	Low	Low	Some
Lin and Fu (52)	Some	Unknown	Low	Low	Low	Some
Liu (53)	Low	High	High	Low	Low	High
Liu et al. (54)	Low	Low	Low	Low	Low	Low
Liu et al. (55)	Some	Low	Low	Low	Low	Some
Liu (56)	Some	Low	Low	Low	Low	Some
Liu et al. (19)	Low	Low	Low	Low	Low	Low
Liu and Zhong (57)	Low	Low	Low	Low	Low	Low
Liu et al. (58)	Low	Low	Low	Low	Low	Low
Lu et al. (59)	Some	Low	Low	Low	Low	Some
Lu et al. (60)	Low	Low	Low	Low	Low	Low
Lu (61)	Some	Low	Low	Low	Low	Some
Lu (62)	Low	Low	Low	Low	Low	Low
Ma et al. (63)	Low	Low	Low	Low	Low	Low
Ma et al. (64)	Low	Low	High	Low	Low	High
Ma et al. (65)	Low	Low	Low	Low	Low	Low
Nie and Huang (66)	Some	Low	Low	Low	Low	Some
Niu (67)	Some	Low	Low	Low	Low	Some
Nahaxibai and Awukaitayi (68)	Low	Low	Low	Low	Low	Low
Liao et al. (69)	Some	Low	Low	Low	Low	Some
Peng et al. (70)	Some	Low	Low	Low	Low	Some
Qian (71)	Some	Low	Low	Low	Low	Some
Ren et al. (72)	Some	High	Low	Low	Low	High
Shen (12)	Some	Low	Low	Low	Low	Some
Shen (73)	Low	Low	Low	Low	Low	Low
Situ et al. (74)	Some	High	Low	Low	Low	High
Chen and Sun (75)	Some	Low	Low	Low	Low	Some
Tong and Zhong (76)	Low	Low	Low	Low	Low	Low
Wan and Tao (15)	Low	Low	Low	Low	Low	Low
Wang et al. (77)	Some	Low	Low	Some	Low	Some
Wang et al. (78)	Some	Low	Low	Some	Low	Some
Wang (17)	Low	Low	Low	Low	Low	Low
Wang et al. (13)	Low	Low	Low	Low	Low	Low
Wang et al. (79)	Some	Low	Low	Some	Low	Some
Wang and Wang (80)	Some	Low	Low	Some	Low	Some
Wang and Bo (81)	Some	Low	Low	Some	Low	Some
Wang and Chen (14)	Some	Low	Low	Some	Low	Some
Wang et al. (82)	Low	High	Low	Some	Low	High
Wang (83)	Some	Low	Low	Some	Low	Some
Wang et al. (84)	Some	Low	Low	Some	Low	Some
Wang and Wang (16)	Some	Low	Low	Some	Low	Some
Wei (85)	Some	Low	Low	Some	Low	Some
Wu (86)	Some	Low	High	Some	Low	High
Wu and Miao (87)	Low	Low	Low	Some	Low	Some
Xiang and Jin (88)	Some	Low	Low	Some	Low	Some
Xu et al. (89)	Some	Low	Low	Some	Low	Some
Xu and Dai (90)	Low	Low	Low	Some	Low	Some
Xu et al. (91)	Some	Low	Low	Some	Low	Some
Yang et al. (11)	Some	Low	Low	Some	Low	Some
Yang et al. (92)	Low	High	Low	Some	Low	High
Yang et al. (93)	Some	Low	Low	Some	Low	Some
Yang and Chen (94)	Some	Low	Low	Some	Low	Some
Yao (95)	Low	Low	Low	Some	Low	Some
Yao et al. (96)	Some	Low	Low	Some	Low	Some
Yao et al. (20)	Some	Low	Low	Some	Low	Some
Ye and Wu (97)	Low	Low	Low	Low	Low	Low
Yin et al. (98)	Low	Low	Low	Low	Low	Low
You et al. (99)	Low	Low	Low	Low	Low	Low
Yu et al. (100)	Low	Low	Low	Low	Low	Low
Wang and Zhang (101)	Low	Low	Low	Low	Low	Low
Zhang (102)	Some	Low	Low	Some	Low	Some
Zhang and Ye (103)	Some	Low	Low	Some	Low	Some
Zhang and Ren (104)	Some	High	Low	Some	Low	High
Zhang et al. (105)	Some	Low	Low	Some	Low	Some
Zhang (106)	Low	Low	Low	Low	Low	Low
Zhang and Jiang (107)	Low	Low	Low	Low	Low	Low
Zhang et al. (108)	Some	Low	Low	Low	Low	Some
Zhang (109)	Low	Low	Low	Low	Low	Low
Yu (110)	Some	Low	Low	Low	Low	Some
Xiamier et al. (111)	Some	Low	Low	Low	Low	Some
Zhu et al. (112)	Low	Low	Low	Low	Low	Low
Zuo and Zhang (113)	Low	Low	Low	Low	Low	Low
Chen et al. (114)	Some	Low	Low	Some	Low	Some
Shang (115)	Some	Low	Low	Some	Low	Some
Pan and Wei (116)	Some	Low	Low	Some	Low	Some
Li et al. (117)	Low	Low	Low	Low	Low	Low

### 3.7 Assessment of consistency test

For the consistency evaluation between direct and indirect evidence, this study conducted a consistency test using network meta-analysis based on frequentist principles. The results of the consistency model calculation showed that direct evidence and indirect evidence had high consistency in all comparisons, meaning no significant statistical differences were observed between results from different research sources. In terms of heterogeneity analysis, this study used the τ^2^ statistic to quantify the heterogeneity among different studies. According to established criteria, most comparisons in this study showed τ^2^ values at low to moderate levels, indicating small differences between studies and relatively robust research results (see Table 3). On the other hand, funnel plots generated from data corresponding to the 7 included outcome indicators showed good symmetry, with no obvious bias observed.

**TABLE 3:** Assessment of consistency and heterogeneity of outcome indicators.

Outcome indicators		Consistency analysis		Heterogeneity(τ^2^)
		Chi^2^	*P*	
Total effective rate		2.29	0.1303	0.00110
VAS score		4.97	0.2059	0.03273
Sex hormone-related indicators	E2	4.47	0.3146	0.03356
	FSH	0.62	0.4327	0.23520
	LH	3.68	0.5521	0.06321
Carbohydrate antigen 125 (CA125)		3.38	0.6539	0.07622
Diameter of ectopic cyst		2.83	0.1725	0.18166
Recurrence rate		1.22	0.3518	0.02096
Adverse reactions		0.03	0.8533	0.24741

### 3.8 Network meta-analysis results of primary outcome indicators

#### 3.8.1 Network evidence graph of interventions

**FIGURE 3:**
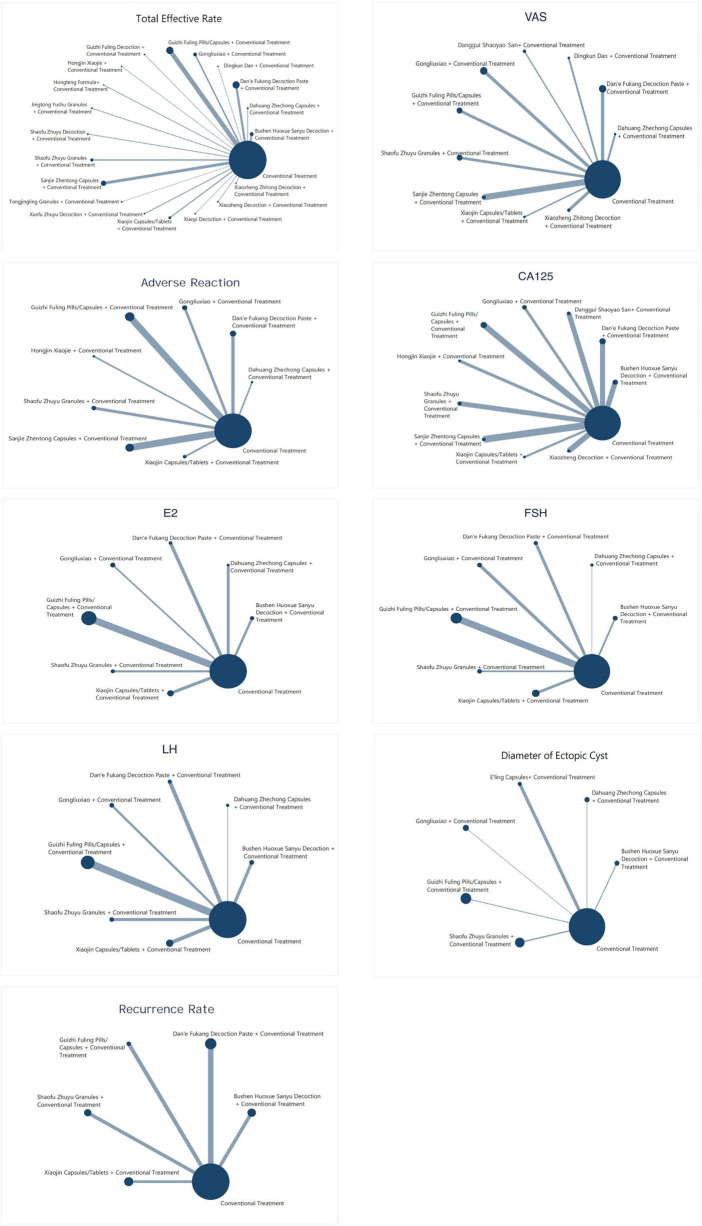
Evidence network relationship graph of interventions.

To intuitively display the direct comparison relationships and evidence strength between different interventions, this study plotted network evidence graphs corresponding to the primary outcome indicators (see Figure 3). In the graph, each node represents an intervention, and the size of the node reflects the sample size of the studies included for that intervention. The thickness of the connecting lines indicates the number of studies involved in the comparison and the strength of direct evidence.

From the network structure, it can be seen that most interventions are centered around conventional biomedicine treatment. CCPPs or compound formulas combined with biomedicine, as common intervention methods, have participated in the comparison of multiple outcome indicators. Overall, the network evidence graph reflects the extensive application and solid research foundation of blood-activating and stasis-resolving drugs and their combined therapies in the intervention of endometriosis.

#### 3.8.2 Total effective rate

**FIGURE 4:**
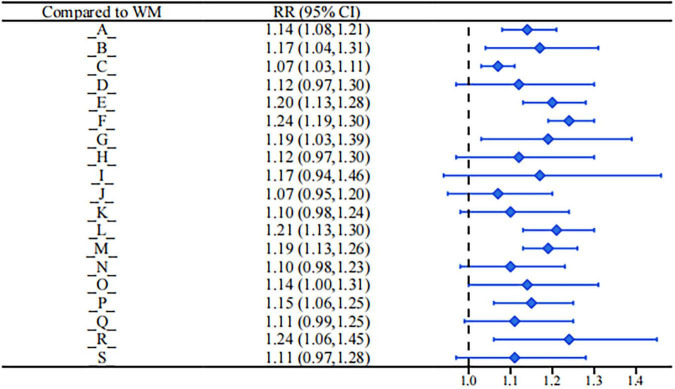
Forest plot of total effective rate.

This study used network meta-analysis to compare the relative effects of various blood-activating and stasis-resolving drug-related interventions on improving the total effective rate in patients with blood stasis syndrome, pain, and discomfort symptoms. The results showed that multiple traditional Chinese medicine preparations and combined conventional treatment interventions were superior to conventional biomedicine treatment (see Figure 4).

Taking conventional clinical medicine treatment as the control, the following interventions demonstrated a significantly higher total effective rate: Bushen Huoxue Sanyu Decoction (RR = 1.14, 95%CI: 1.08, 1.21), Dahuang Zhechong Capsule (RR = 1.17, 95%CI: 1.04, 1.31). Dan’e Fukang Decoction Extract (RR = 1.07, 95%CI: 1.03, 1.11), Gongliuxiao Capsule (RR = 1.20, 95%CI: 1.13, 1.28), Guizhi Fuling Pill/Capsule (RR = 1.24, 95%CI: 1.19, 1.30), Guizhi Fuling Decoction (RR = 1.19, 95%CI: 1.03, 1.39), Shaofu Zhuyu Granule (RR = 1.21, 95%CI: 1.13, 1.30). Sanjie Zhentong Capsule (RR = 1.19, 95%CI: 1.13, 1.26), Xuefu Zhuyu Decoction (RR = 1.14, 95%CI: 1.00, 1.31), Xiaojin Capsule/Tablet (RR = 1.15, 95%CI: 1.06, 1.25), Xiaozheng Decoction (RR = 1.24, 95%CI: 1.06, 1.45).

In comparisons between different drug interventions: Bushen Huoxue Sanyu Decoction was superior to Dan’e Fukang Decoction Extract in improving the total effective rate (RR = 1.07, 95%CI: 0.74, 0.93). Dan’e Fukang Decoction Extract was superior to Gongliuxiao Capsule (RR = 0.89, 95%CI: 0.82, 0.96) and also superior to Guizhi Fuling Pill/Capsule (RR = 0.86, 95%CI: 0.81, 0.91). Guizhi Fuling Pill/Capsule, in turn, was superior to Jingtong Yushu Granule (RR = 1.16, 95%CI: 1.02, 1.32) and Tongjingling Granule (RR = 1.13, 95%CI: 1.00, 1.27). No statistical differences were observed in comparisons between the remaining interventions.

#### 3.8.3 VAS score

**FIGURE 5:**
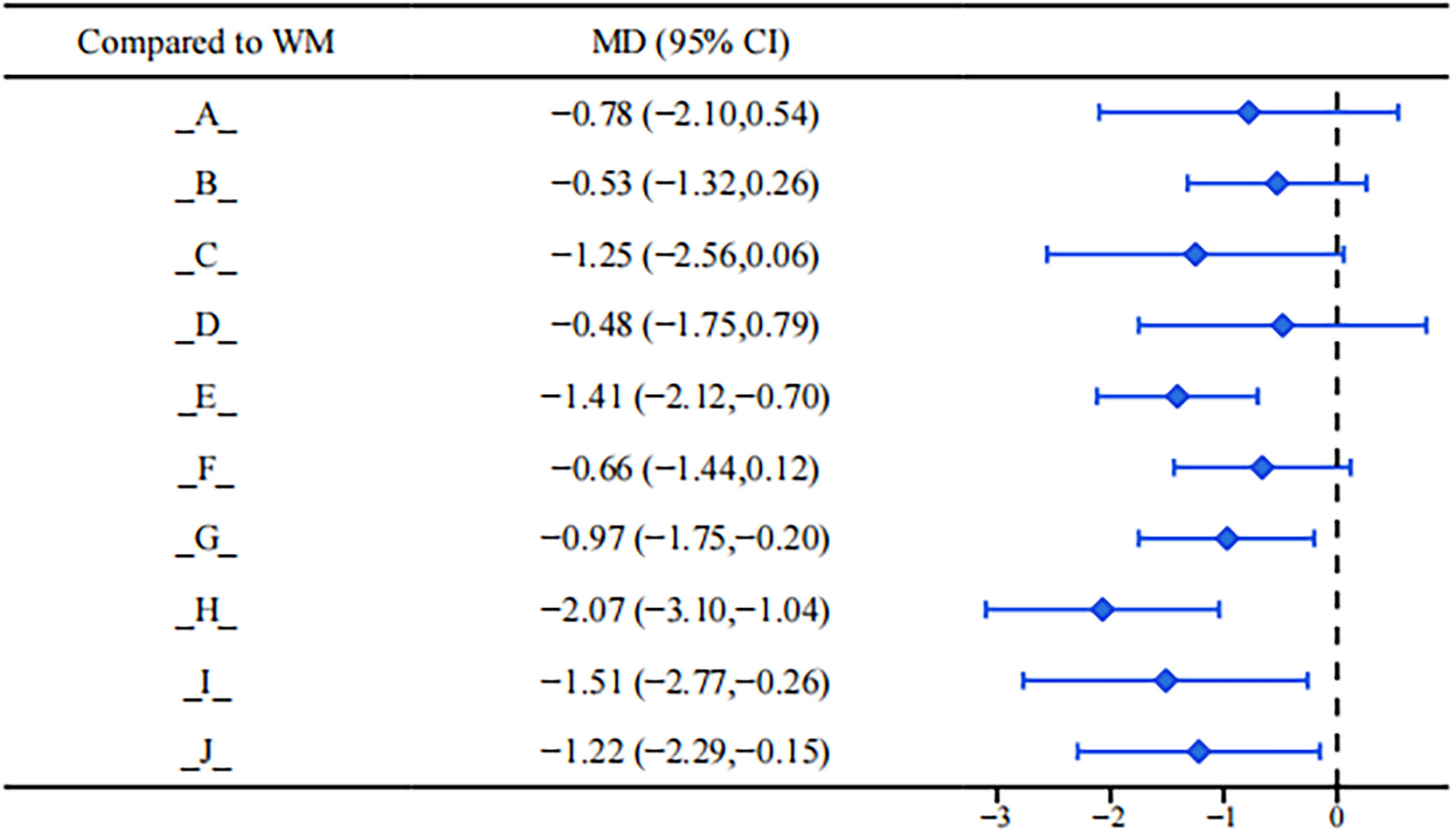
Forest plot of VAS score.

Results of the Visual Analog Scale (VAS) showed that various blood-activating and stasis-resolving drugs combined with conventional clinical medicine therapy had more advantages in reducing VAS scores compared with conventional clinical medicine alone (see Figure 5). Compared with conventional clinical medicine, the following interventions showed statistically significant differences in reducing VAS scores: Gongliuxiao Capsule (MD = −1.41, 95%CI: −2.12, −0.70). Shaofu Zhuyu Granule (MD = −0.97, 95%CI: −1.75, −0.20). Sanjie Zhentong Capsule (MD = −2.07, 95%CI: −3.10, −1.04). Xiaojin Capsule/Tablet (MD = −1.51, 95%CI: −2.77, −0.26). Xiaozheng Zhitong Decoction (MD = −1.22, 95%CI: −2.29, −0.15). All mean differences (MD) were less than 0, suggesting that the above interventions could significantly reduce patients’ VAS scores and improve dysmenorrhea symptoms.

#### 3.8.4 Sex hormone-related indicators [estradiol (E2), follicle-stimulating hormone (FSH), luteinizing hormone (LH)]

##### 3.8.4.1 Estradiol (E2)

**FIGURE 6:**
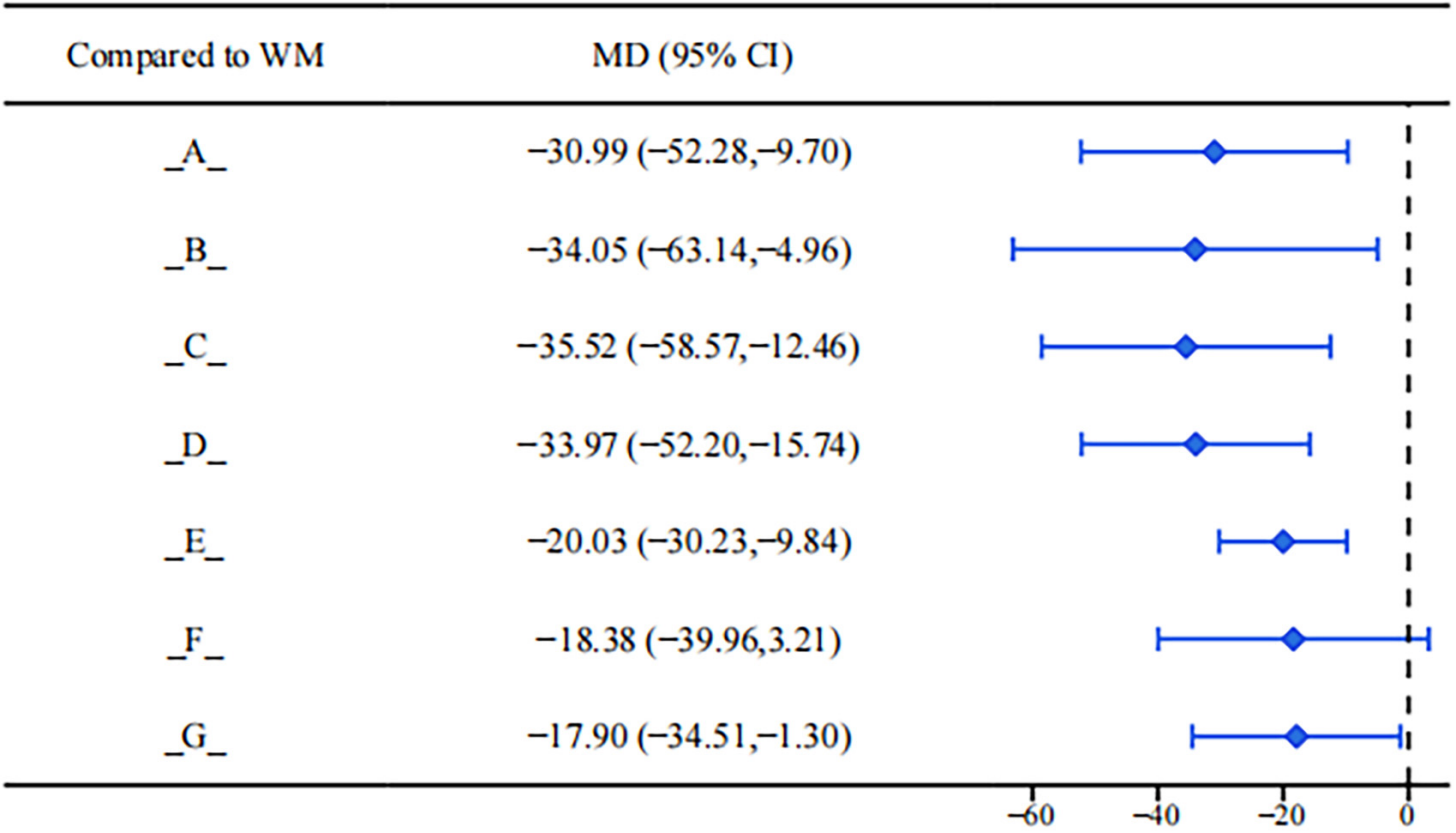
Forest plot of E_2_.

Results of the league table for estradiol (E2) showed that, compared with conventional clinical medicine treatment, the following interventions had more significant clinical advantages in reducing E2 levels (see Figure 6): Bushen Huoxue Sanyu Decoction (MD = −30.99, 95%CI: −52.28, −9.70). Dahuang Zhechong Capsule (MD = −34.05, 95%CI: −63.14, −4.96). Dan’e Fukang Decoction Extract (MD = −35.52, 95%CI: −58.57, −12.46). Gongliuxiao Capsule (MD = −33.97, 95%CI: −52.20, −15.74). Guizhi Fuling Pill/Capsule (MD = −20.03, 95%CI: −30.23, −9.84). Xiaojin Capsule/Tablet (MD = −17.90, 95%CI: −34.51, −1.30). The remaining interventions showed a certain trend of improvement but did not reach a statistically significant level.

##### 3.8.4.2 Follicle-stimulating hormone (FSH)

**FIGURE 7:**
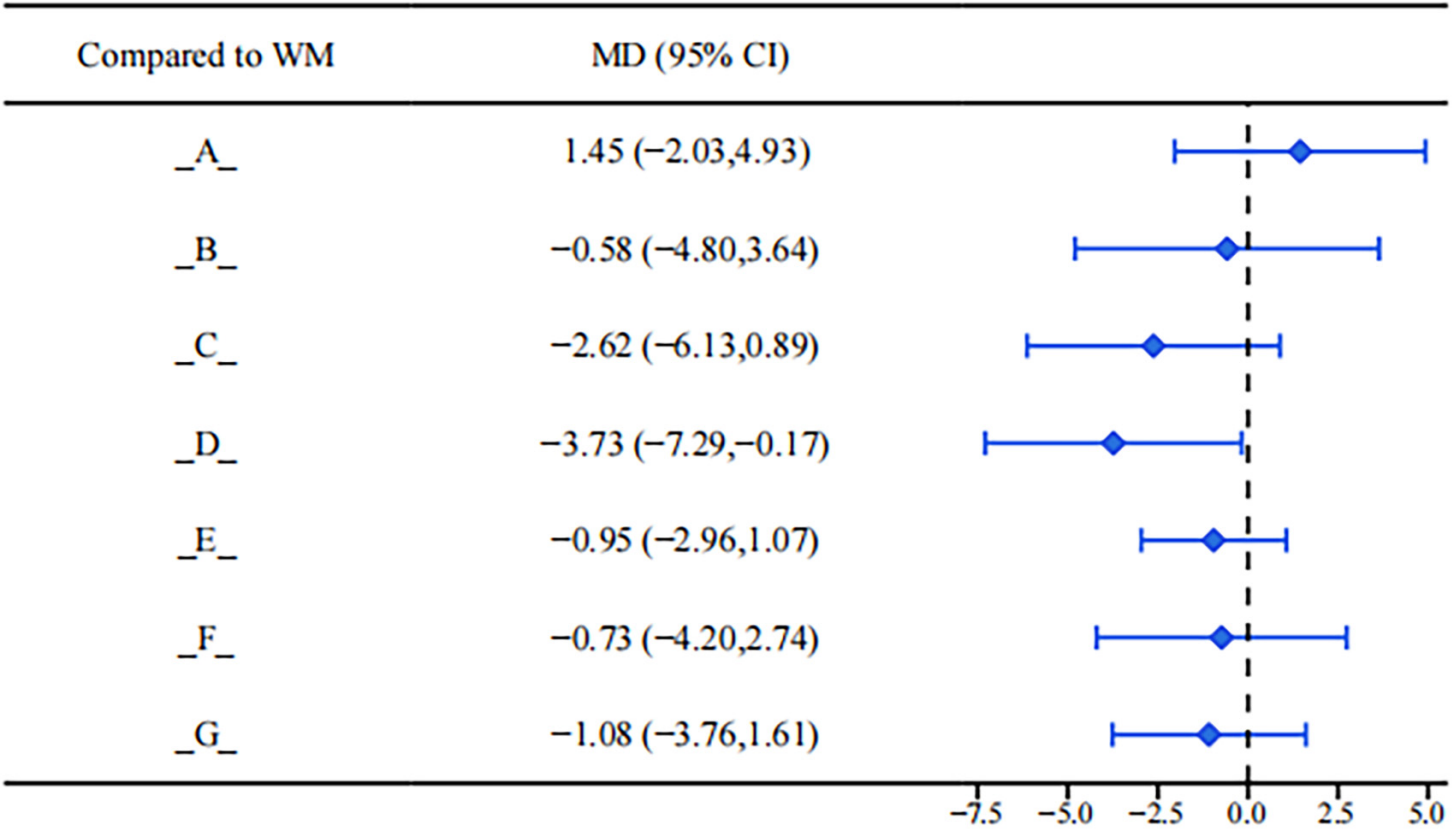
Forest plot of FSH.

Abnormal levels of follicle-stimulating hormone (FSH) often reflect ovarian reserve dysfunction or feedback imbalance of the reproductive endocrine system. Data from the league table showed that (see Figure 7), compared with conventional clinical medicine treatment, only Gongliuxiao Capsule showed a potential advantage in reducing FSH levels and stabilizing ovarian function (MD = −3.73, 95%CI: −7.29, −0.17). Although other interventions showed a downward trend in FSH, the statistical significance was insufficient, and further evaluation of their actual clinical value is required by combining mechanism-based research and long-term follow-up.

##### 3.8.4.3 Luteinizing hormone (LH)

**FIGURE 8:**
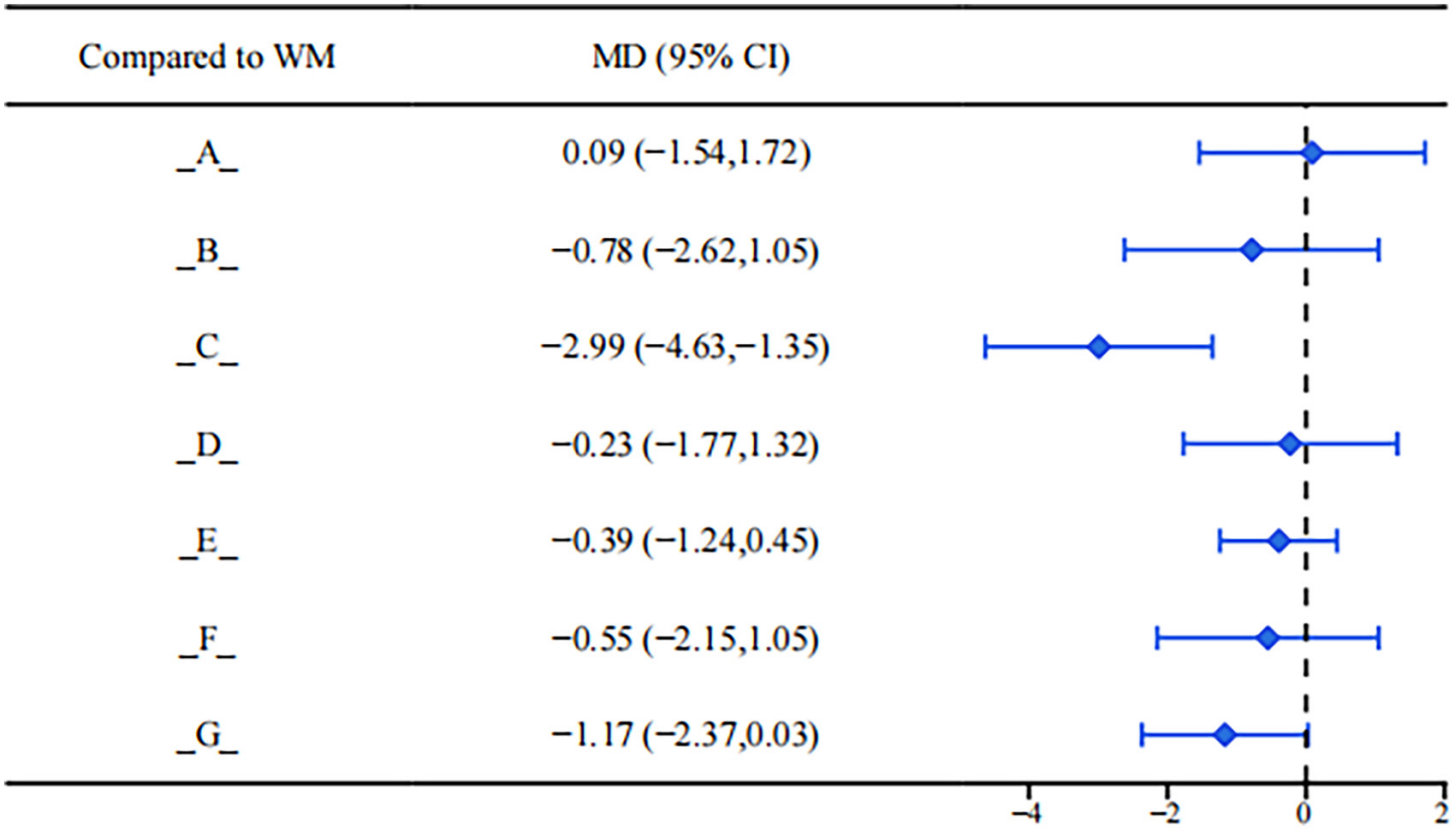
Forest plot of LH.

In patients with endometriosis, elevated levels of luteinizing hormone (LH) may indicate dysfunction of the hypothalamic-pituitary-ovarian axis, affecting ovulation regularity and estrogen feedback regulation. Results from the league table (see Figure 8) showed that Dan’e Fukang Decoction Extract was superior to conventional biomedicine treatment (MD = −2.99, 95%CI: −4.63, −1.35). Comparisons between interventions indicated that Dan’e Fukang Decoction Extract demonstrated better efficacy in reducing LH levels than the following interventions: Gongliuxiao Capsule (MD = −2.76, 95%CI: −5.01, −0.51), Guizhi Fuling Pill/Capsule (MD = −2.59, 95%CI: −4.44, −0.75), Shaofu Zhuyu Granule (MD = −2.44, 95%CI: −4.73, −0.15). This may help restore LH rhythm and improve ovulatory function. Although the remaining interventions showed a certain numerical downward trend in LH levels, the 95% confidence intervals crossed zero, suggesting insufficient statistical evidence for their efficacy in regulating LH. Further verification of the stability of their intervention effects through subsequent studies is required.

#### 3.8.5 CA125

**FIGURE 9:**
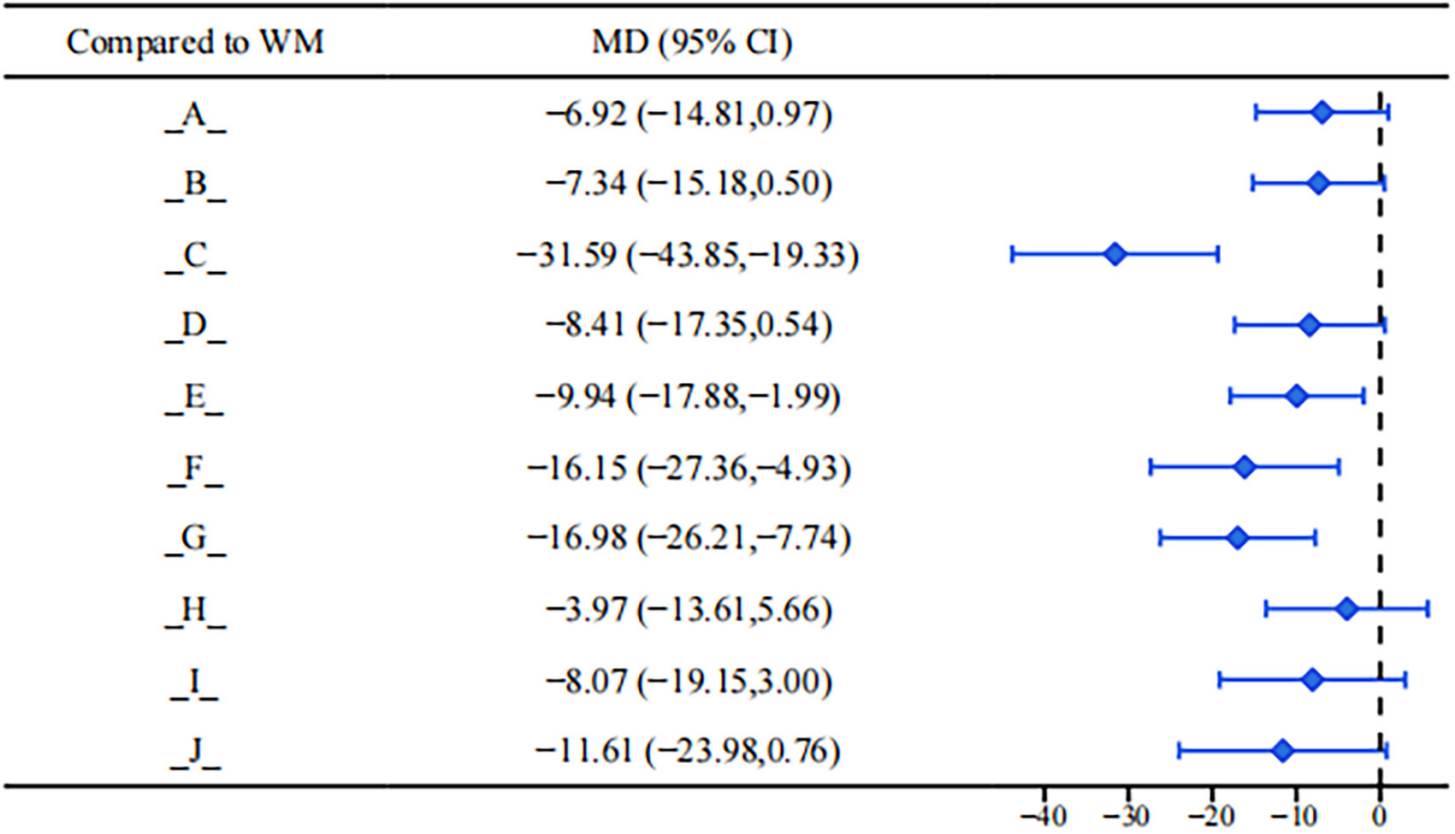
Forest plot of CA125.

In the network meta-analysis of CA125 levels, multiple traditional Chinese medicine interventions showed more significant reducing effects compared with conventional biomedicine. Specifically, the following interventions demonstrated superior efficacy in reducing CA125 levels compared with the control group, with statistically significant differences (see Figure 9): Bushen Huoxue Sanyu Decoction (MD = −1.00, 95%CI: −1.98, −0.03), Danggui Shaoyao Powder (MD = −2.31, 95%CI: −3.69, −0.92), Gongliuxiao Capsule (MD = −1.25, 95%CI: −2.37, −0.13), Guizhi Fuling Pill/Capsule (MD = −1.14, 95%CI: −2.11, −0.17), Hongjin Xiaojie Capsule (MD = −1.60, 95%CI: −2.99, −0.22)

Shaofu Zhuyu Granule (MD = −1.57, 95%CI: −2.69, −0.44), Xiaozheng Decoction (MD = −1.55, 95%CI: −2.93, −0.17). This suggests they may have a more definite effect in regulating endometriosis-related inflammatory responses. In contrast, although some other interventions also showed a downward trend in CA125 in the league table, their 95% confidence intervals crossed zero, indicating high uncertainty in efficacy. Current evidence is insufficient to support their stable advantage in reducing CA125 levels.

#### 3.8.6 Diameter of ectopic cyst

**FIGURE 10:**
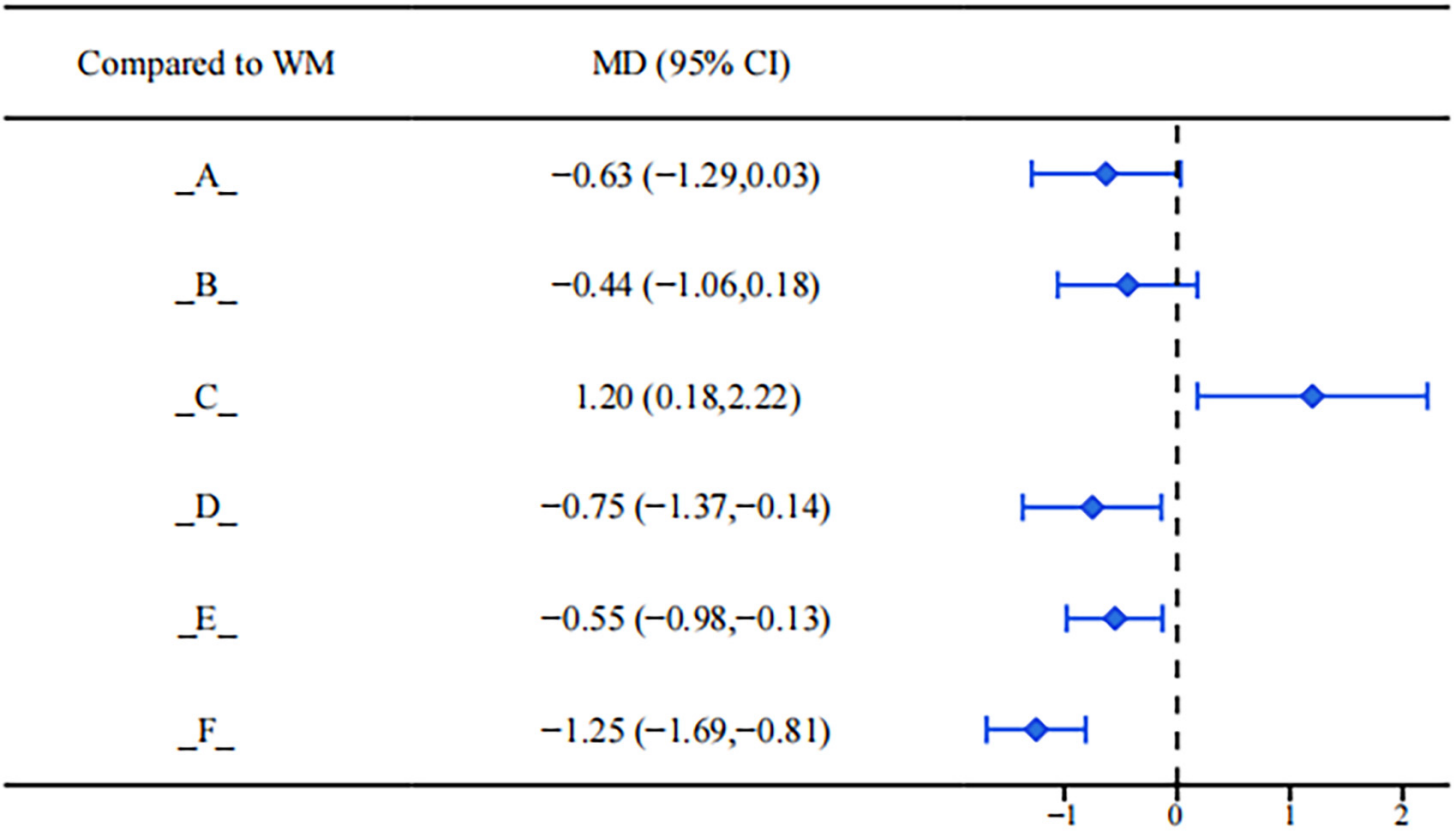
Forest plot of ectopic cyst diameter.

In the network meta-analysis, there were certain differences in the degree of influence of various interventions on the diameter of ectopic cysts (see Figure 10). Among them, Gongliuxiao Capsule (MD = −0.75, 95%CI: −1.37, −0.14), Guizhi Fuling Pill/Capsule (MD = −0.55, 95%CI: −0.98, −0.13), and Shaofu Zhuyu Granule (MD = −1.25, 95%CI: −1.69, −0.81) showed significant advantages over conventional biomedicine treatment alone in reducing cyst diameter. Their mean differences were negative, and the 95% confidence intervals did not cross zero, suggesting statistically significant efficacy. This may be achieved by inhibiting the growth of ectopic lesions and promoting their absorption. Although other interventions showed a certain trend of cyst reduction in some studies, the current statistical effects are not yet stable. Due to the wide confidence intervals of their effect estimates or crossing the null line, it is still difficult to clearly determine whether their intervention effects are superior to conventional treatment.

#### 3.8.7 Recurrence rate

**FIGURE 11:**
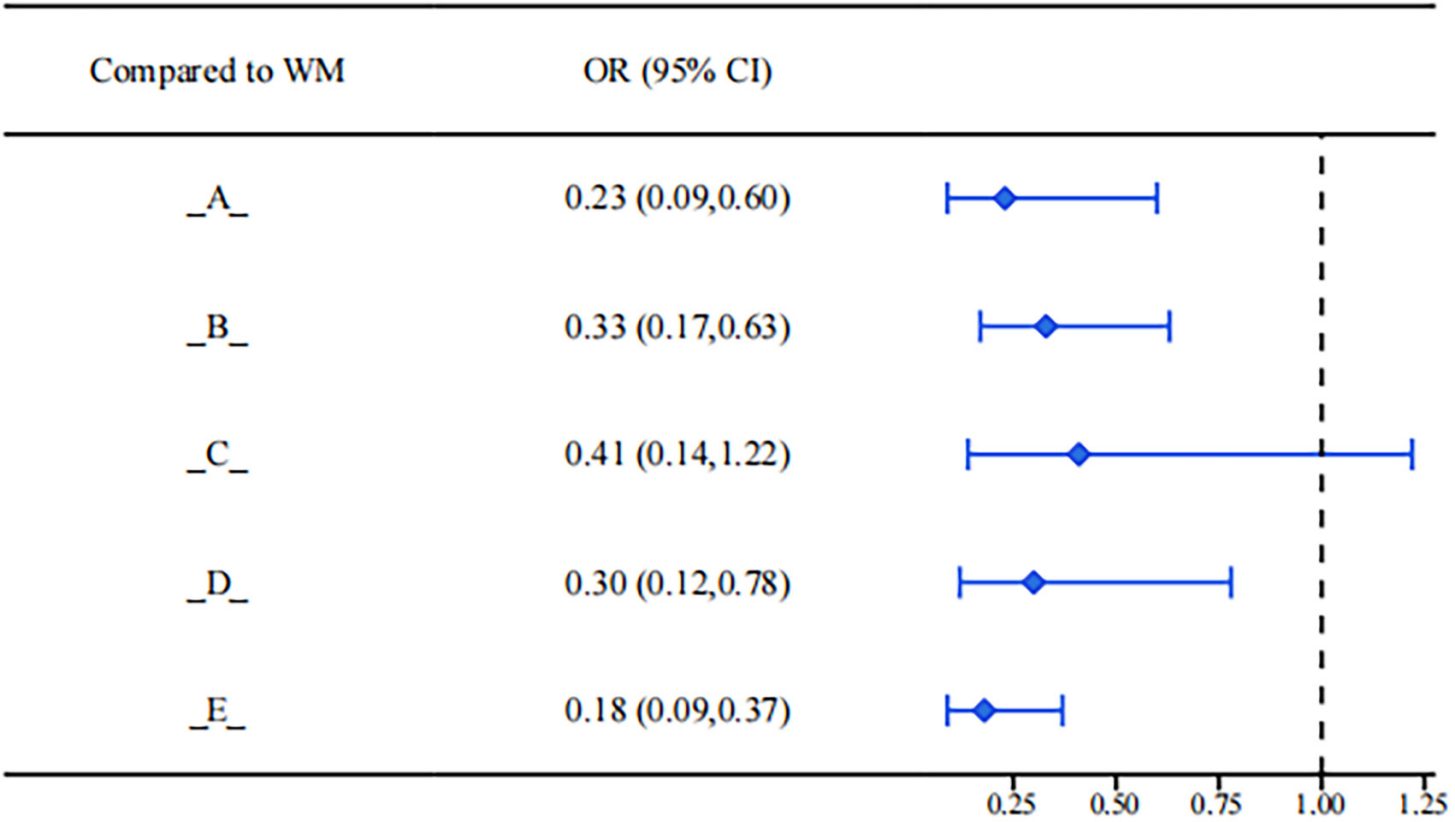
Forest plot of recurrence rate.

Regarding the control of endometriosis recurrence, different interventions showed certain differences in reducing the recurrence rate. Results from the league table (see Figure 11) indicated that Bushen Huoxue Sanyu Decoction (OR = 0.23, 95%CI: 0.09, 0.60), Dan’e Fukang Decoction Extract (OR = 0.33, 95%CI: 0.17, 0.63), Shaofu Zhuyu Granule (OR = 0.30, 95%CI: 0.12, 0.78), and Xiaojin Capsule/Tablet (OR = 0.18, 95%CI: 0.09, 0.37) had significant advantages over conventional biomedicine in reducing the recurrence risk. All effect values were less than 1, and the confidence intervals did not cross the null line, suggesting that they may achieve more effective disease control by improving the internal environment or inhibiting lesion regeneration.

#### 3.8.8 Incidence of adverse reactions

**FIGURE 12:**
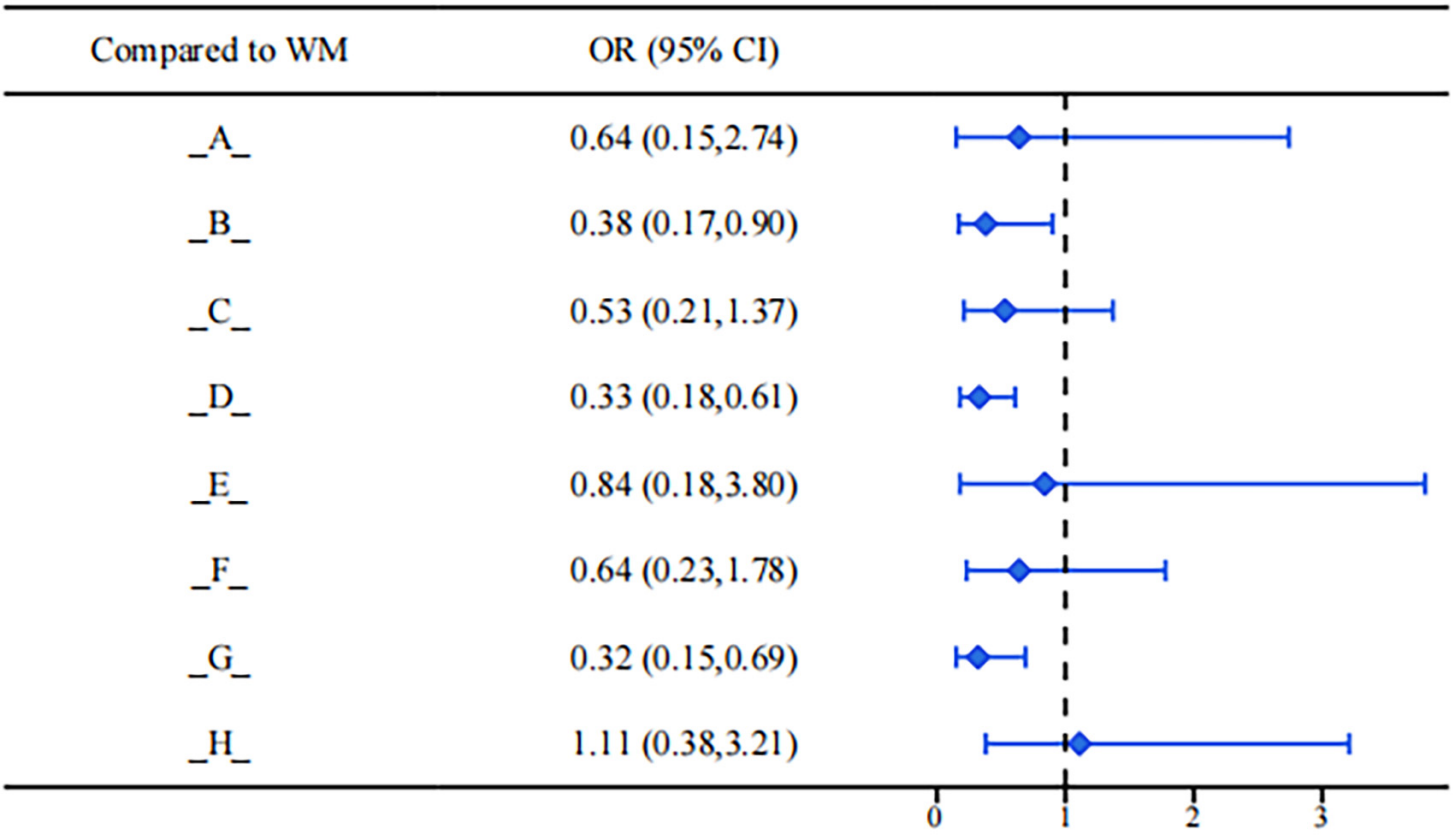
Forest plot of adverse reactions.

In the comparison of adverse reaction incidence, different interventions showed certain differences in terms of safety performance. According to the results of the league table analysis (see Figure 12), Dan’e Fukang Decoction Extract (OR = 0.38, 95%CI: 0.17, 0.90), Guizhi Fuling Pill/Capsule (OR = 0.33, 95%CI: 0.18, 0.61), and Sanjie Zhentong Capsule (OR = 0.32, 95%CI: 0.15, 0.69) demonstrated better tolerability and favorable safety profiles compared with conventional medicine in reducing the incidence of adverse reactions. Although other interventions also showed a certain trend of improvement in adverse reaction control, their statistical effects did not reach significance, or their confidence intervals were relatively wide. Therefore, in clinical practice, a comprehensive evaluation of efficacy and safety is still required for individualized assessment.

#### 3.8.9 SUCRA probability ranking

**FIGURE 13:**
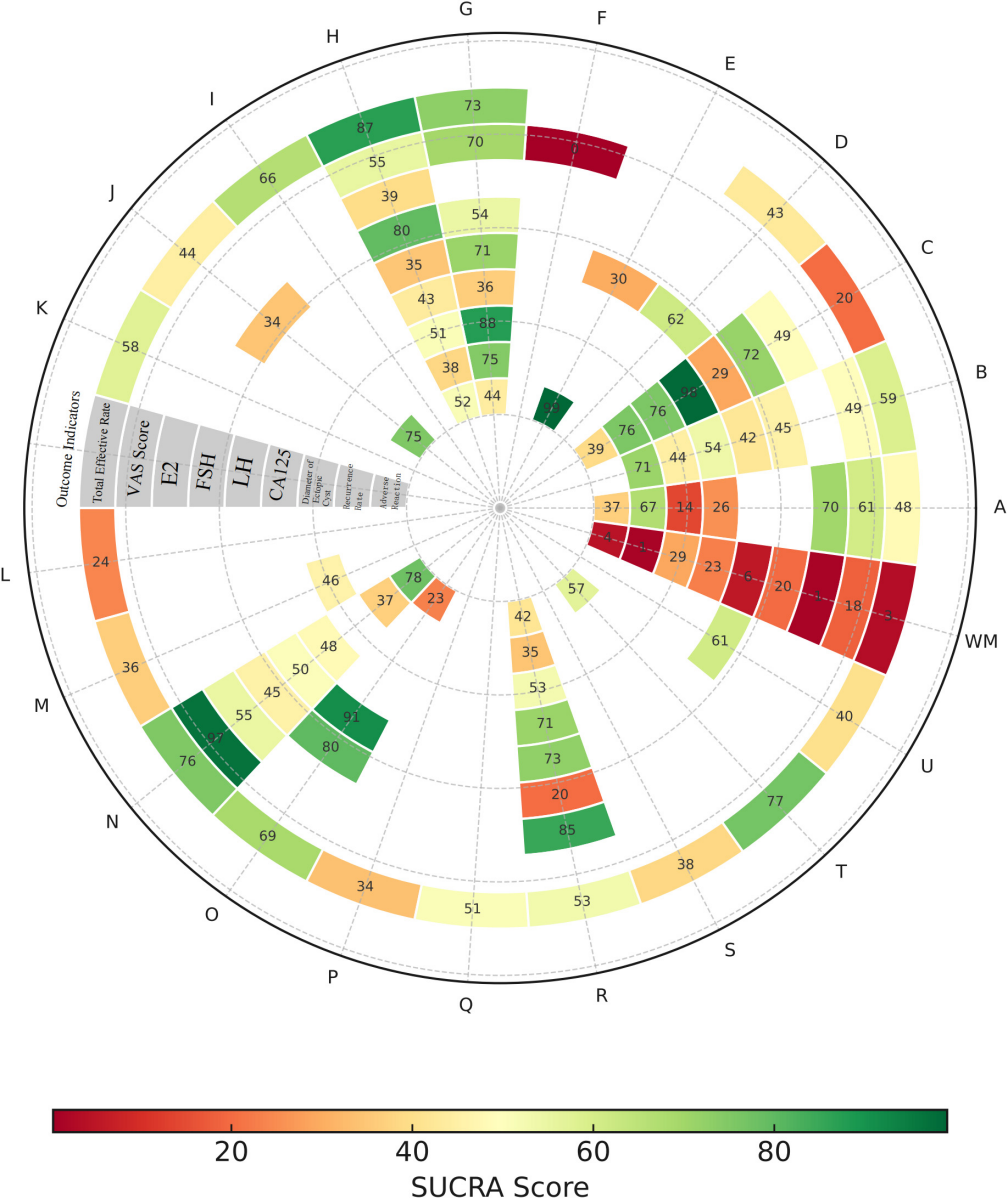
Heatmap of SUCRA probability ranking. Interventions: A: Bushen Huoxue Sanyu Decoction + biomedicine treatment. B: Dahuang Zhechong Capsule + biomedicine treatment. C: Dan’e Fukang Decoction Extract + biomedicine treatment. D: Dingkun Dan + biomedicine treatment. E: Danggui Shaoyao Powder + biomedicine treatment. F: Eleng Capsule + biomedicine treatment. G: Gongliuxiao + biomedicine treatment. H: Guizhi Fuling Pill/Capsule + biomedicine treatment. I: Guizhi Fuling Decoction + biomedicine treatment. J: Hongjin Xiaojie Capsule + biomedicine treatment. K: Hongteng Formula + biomedicine treatment. L: Jingtong Yushu Granule + biomedicine treatment. M: Shaofu Zhuyu Decoction + biomedicine treatment. N: Shaofu Zhuyu Granule + biomedicine treatment. O: Sanjie Zhentong Capsule + biomedicine treatment. P: Tongjingling Granule + biomedicine treatment. Q: Xuefu Zhuyu Decoction + biomedicine treatment. R: Xiaojin Capsule/Tablet + biomedicine treatment. S: Xiaoyi Decoction + biomedicine treatment. T: Xiaozheng Decoction + biomedicine treatment. U: Xiaozheng Zhitong Decoction + biomedicine treatment. V: Conventional biomedicine therapy + biomedicine treatment.

To comprehensively evaluate the efficacy ranking of various interventions across different outcome indicators, this study used SUCRA (Surface Under the Cumulative Ranking) values for comparison. A higher SUCRA value indicates superior efficacy of the intervention for that indicator. The results of the SUCRA analysis for outcome indicators are as follows (see Figure 13):

In terms of total effective rate, the top-ranked interventions by SUCRA values were Guizhi Fuling Pill/Capsule + Biomedicine (87.4), Shaofu Zhuyu Granule + Biomedicine (76.8), and Xiaozheng Decoction + Biomedicine (75.5), indicating that integrated traditional Chinese and biomedicine therapy has greater advantages in improving overall clinical efficacy.

For VAS score, interventions with higher scores included Sanjie Zhentong Capsule + Biomedicine (91.3), Xiaojin Capsule/Tablet + Biomedicine (72.8), and Gongliuxiao Capsule + Biomedicine (71.3), suggesting that combined therapy has favorable effects in relieving pain symptoms.

In reducing E2 levels, Dan’e Fukang Decoction Extract + Biomedicine (75.9), Gongliuxiao Capsule + Biomedicine (74.6), and Dahuang Zhechong Capsule + Biomedicine (71.3) ranked among the top, further supporting their stable and superior efficacy in regulating hyperestrogenic states.

SUCRA ranking showed that Gongliuxiao Capsule + Biomedicine (87.6), Dan’e Fukang Decoction Extract + Biomedicine (75.6), and Xiaojin Capsule/Tablet (52.6) also achieved high rankings in reducing FSH levels. This indicates they may be more effective in improving ovarian dysfunction and regulating pituitary feedback, especially suitable for patient populations with abnormally elevated FSH.

In the SUCRA ranking for LH, Dan’e Fukang Decoction Extract + Biomedicine, Xiaojin Capsule/Tablet + Biomedicine, and Dahuang Zhechong Capsule + Biomedicine took the top positions with SUCRA values of 98.4, 71, and 54.1% respectively, suggesting these interventions may have excellent effects in balancing LH secretion and improving ovulation regulation.

For the serum marker CA125, Danggui Shaoyao Powder, Shaofu Zhuyu Granule, and Hongjin Xiaojie Capsule led the SUCRA ranking with values of 99.1, 77.9, and 75.2% respectively. This indicates they have a higher comprehensive advantage in reducing CA125 levels and controlling endometriosis-related inflammatory responses.

In the SUCRA ranking for the diameter of ectopic cysts, Shaofu Zhuyu Granule + Biomedicine (96.7), Gongliuxiao Capsule + Biomedicine (69.5), and Bushen Huoxue Sanyu Decoction + Biomedicine (60.7) ranked high. This result suggests that the above interventions have a higher comprehensive ranking in reducing cyst volume, and their therapeutic effects may be achieved by promoting lesion absorption and improving the pelvic microenvironment.

The SUCRA ranking results for recurrence rate showed that Xiaojin Capsule/Tablet + Biomedicine, Bushen Huoxue Sanyu Decoction + Biomedicine, and Shaofu Zhuyu Granule + Biomedicine took the leading positions in long-term recurrence control, with SUCRA values of 85.2, 70.1, and 55%, respectively. This ranking structure is consistent with the OR values observed in the league table, indicating that these interventions may play a certain role in delaying or reducing the risk of recurrence.

In the SUCRA ranking for the incidence of adverse reactions, Sanjie Zhentong Capsule (80), Guizhi Fuling Pill/Capsule (79.7), and Dan’e Fukang Decoction Extract (71.5) achieved relatively high rankings. This indicates that they have better safety performance and patient tolerability during treatment, or are more suitable for patient populations sensitive to drug reactions or with complex underlying conditions.

In summary, through the analysis of efficacy rankings of different interventions across various outcome indicators using SUCRA values, it can be seen that integrated traditional Chinese and biomedicine regimens exhibit prominent therapeutic advantages in multiple dimensions. Especially in key efficacy indicators such as total effective rate, VAS score, regulation of hormone levels (E2, FSH, LH), and reduction of CA125, combined interventions such as Guizhi Fuling Pill + Biomedicine, Dan’e Fukang Decoction Extract + Biomedicine, Shaofu Zhuyu Granule + Biomedicine, and Gongliuxiao Capsule + Biomedicine consistently rank high. This suggests that they have favorable comprehensive effects in improving symptoms, regulating endocrine function, and inhibiting lesion activity. The SUCRA ranking results not only confirm the multiple efficacy advantages of combined interventions but also provide a reliable basis for the selection of clinical individualized medication strategies.

### 3.9 Publication bias analysis

**FIGURE 14:**
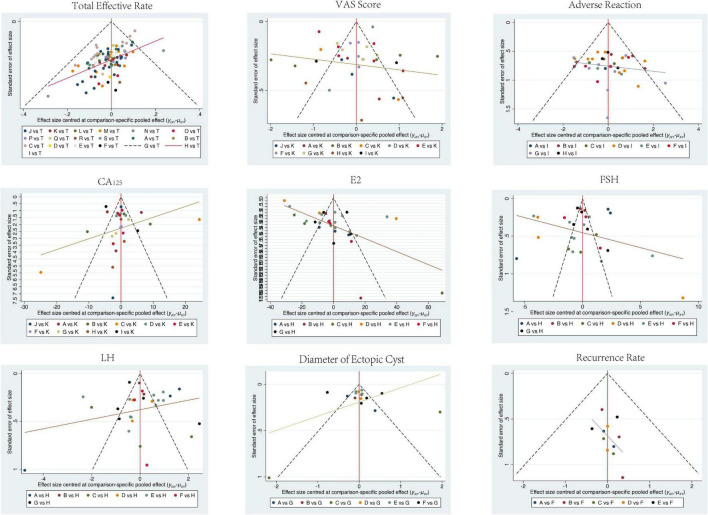
Funnel plot.

To evaluate the publication bias and small-sample effects of the included studies, this research plotted comparison-adjusted funnel plots for the primary outcome indicators (total effective rate, VAS score, E2, FSH, LH, CA125, diameter of ectopic cyst, recurrence rate, and adverse reactions) (see Figure 14). The distribution of points in the funnel plots reflects the distribution of effect sizes and standard errors across studies; good symmetry typically indicates a low risk of publication bias.

From the graphical results, the funnel plots for most outcome indicators showed a relatively balanced distribution with good central symmetry. No obvious clustering of small-sample studies on either side was observed, and the overall structure appeared well-symmetrical. This suggests that the included studies generally have a low risk of publication bias.

## 4 Discussion

Endometriosis is a common and refractory disease among reproductive-aged women, characterized by a high incidence and frequent recurrence. Biomedicine treatment mainly relies on hormonal drug suppression and surgical intervention; however, hormonal treatments are associated with numerous side effects during the course of therapy, while surgery is difficult to achieve a radical cure and is accompanied by a high postoperative recurrence rate. Patients gain limited long-term benefits, so there is an urgent need to explore better strategies to relieve symptoms, reduce recurrence, and minimize adverse reactions. Against this backdrop, integrated traditional Chinese and biomedicine therapy has attracted attention due to its advantages of emphasizing both holistic regulation and rapid intervention, holding the promise of compensating for the shortcomings of single-modality treatments.

The network meta-analysis of this study showed that: in terms of core outcomes such as total effective rate, pain relief (VAS score), and hormone levels, integrated traditional Chinese and biomedicine regimens were significantly superior to biomedicine treatment alone. Combined medication significantly improved the overall clinical effective rate, meaning that more patients achieved effective control of symptoms; at the same time, it effectively reduced the VAS score, suggesting more adequate relief of pain symptoms such as dysmenorrhea. In terms of endocrine regulation, compared with single biomedicine, the combined therapy more effectively reduced the excessively high estradiol (E2) levels in patients and exerted a certain regulatory effect on abnormally elevated FSH and LH, thereby partially restoring the functional balance of the hypothalamic-pituitary-ovarian axis. Notably, the integrated traditional Chinese and biomedicine therapy also showed advantages in safety: the incidence of adverse reactions in some combined groups was significantly lower than that in the control group, indicating that the inclusion of traditional Chinese medicine can alleviate the side effects of biomedicine and improve patient tolerability. In summary, multi-dimensional evidence supports that integrated traditional Chinese and biomedicine has comprehensive advantages in improving symptoms, regulating endocrine dysfunction, and reducing treatment-related side effects.

The improved efficacy of integrated Chinese herbal medicines and biomedicine stems from the complementarity and synergy of the two medical systems in their mechanisms of action. From the perspective of TCM theory, blood stasis obstruction is the core pathogenesis of endometriosis, shared across common syndrome types including kidney deficiency with blood stasis, qi stagnation and blood stasis, and cold coagulation and blood stasis. Accordingly, activating blood circulation and resolving stasis serves as the fundamental therapeutic principle, with all included herbal medicines targeting this shared root pathogenesis regardless of variant accompanying patterns. Modern research has confirmed that such traditional Chinese botanical drugs can reduce blood viscosity, inhibit platelet aggregation and thrombosis, and dilate blood vessels to increase local blood flow, thereby alleviating pelvic ischemia-hypoxia and uterine artery spasm (118). Meanwhile, blood-activating drugs can promote the absorption of inflammatory exudates and scar adhesions, and soften lesion tissues, embodying the efficacy of “resolving stasis to eliminate masses. This helps reduce the size of ectopic cysts and alleviate chronic pain caused by tissue adhesions (119).

Tonifying the kidney and regulating the thoroughfare and conception vessels plays an important role in treating endometriosis by balancing endocrine function and enhancing the body’s disease resistance. Patients with endometriosis often develop deficiencies due to prolonged illness, frequently presenting with a constitutional background of kidney qi deficiency and disharmony of the thoroughfare and conception vessels. In TCM theory, the kidney governs reproduction and endocrine function, and deficiency of kidney essence can lead to menstrual disorders, infertility, and recurrent pain. Therefore, many treatment regimens combine blood-activating and stasis-resolving drugs with kidney-tonifying and menstruation-regulating medicines to address both the root cause (ben) and symptoms (biao). Kidney-tonifying botanical drugs (such as Rehmannia glutinosa (Gaertn.) DC. [Orobanchaceae; Rehmanniae radix praeparata], Cornus officinalis Sieb. et Zucc. [Cornaceae; Corni fructus], Curculigo orchioides Gaertn. [Hypoxidaceae; Curculiginis rhizoma], Epimedium brevicornu Maxim. [Berberidaceae; Epimedii folium], etc.), on the one hand, nourish kidney essence and warm the uterus, improving the patient’s overall functional status; on the other hand, modern pharmacological studies have shown that kidney-warming TCM botanical drugs can also influence the hypothalamic-pituitary-ovarian axis and adrenal cortex function, exerting effects similar to endocrine regulation (120). This confirms that kidney-tonifying therapy can improve ovarian reserve function, stabilize hormonal feedback, and fundamentally alleviate the issue of “constitutional deficiency” (ben xu), thereby reducing the tendency for disease recurrence.

The advantages of integrated Chinese herbal medicines and biomedicine in treating endometriosis hold significant clinical practical significance. Firstly, in terms of symptom improvement and quality of life enhancement: through the analgesic and menstruation-regulating effects of traditional Chinese medicine, patients’ pain symptoms—especially dysmenorrhea and chronic pelvic pain—are relieved more thoroughly (121). This not only alleviates the physical suffering of patients but also reduces the psychological stress and negative emotions caused by recurrent pain, thereby significantly improving their quality of life.

Secondly, compared with the “amenorrhea-style” treatment using high-dose hormones alone, integrated traditional Chinese and biomedicine regimens focus more on the balanced regulation of the endocrine environment. With the assistance of Chinese herbal medicines, the required dosage of conventional hormone therapy may be reduced, thereby minimizing excessive suppression of ovarian function. While effectively lowering estrogen levels to control the disease, the combined therapy has less adverse impact on gonadotropins such as FSH and LH (122). Additionally, some kidney-tonifying Chinese botanical drugs can promote the recovery of ovulatory function, which is particularly crucial for patients with fertility requirements. The comprehensive therapy achieves a better balance between inhibiting lesions and maintaining physiological functions. This advantage means that patients’ menstrual and endocrine conditions tend to be more normal during long-term treatment, preserving a certain degree of fertility potential while relieving symptoms.

Furthermore, the recurrence of endometriosis has long been a challenging issue, and integrated traditional Chinese and biomedicine therapy has demonstrated excellent efficacy in this regard. Multiple studies have indicated that adjuvant traditional Chinese medicine conditioning after surgery or the inclusion of TCM interventions in pharmacological treatment can significantly reduce the medium- and long-term recurrence risk of the disease (121, 123). This means that after receiving combined therapy, patients experience longer symptom remission periods, eliminating the need for frequent repeated surgeries or treatment regimen adjustments, thereby reducing both medical and psychological burdens accordingly. In summary, with its comprehensive advantages in pain control, endocrine balance, lesion inhibition, and safety, integrated traditional Chinese and biomedicine therapy provides a more optimized approach to clinical management for endometriosis patients. In clinical practice, these advantages translate into better patient prognoses, higher satisfaction, improved quality of life, and more efficient utilization of medical resources.

Based on recent literature and clinical guidelines, two administration sequences are recommended for Chinese herbal medicines combined with hormonal agents in EMs. Concurrent combined therapy is preferred, in which Chinese herbal medicines and hormones are administered simultaneously for 3–6 months, suitable for moderate-to-severe patients or those at high risk of postoperative recurrence. Sequential combined therapy is an alternative for patients with poor hormonal tolerance or mild conditions: hormones are used first to control acute symptoms, followed by Chinese herbal medicines for long-term maintenance. During treatment, menstrual patterns, sex hormone levels, liver function, and disease markers (e.g., CA125) should be monitored periodically. No severe interactions between Chinese herbal medicines and hormones have been reported, and the combination can enhance efficacy while reducing adverse reactions, supporting its safety and utility in real-world practice.

Although this study preliminarily confirmed the efficacy advantages of various blood-activating and stasis-resolving drugs (including compound formulas and CCPPs) combined with biomedicine in treating endometriosis through a network meta-analysis based on multiple RCTs, several limitations remain. Firstly, there is significant heterogeneity in TCM syndrome types among the included studies. Some studies did not specify the patients’ syndrome types or the basis for syndrome differentiation, resulting in insufficient matching between syndromes and interventions, which may affect the accuracy of explaining the efficacy of TCM. Secondly, TCM interventions are characterized by significant individualization and multi-formulation features. Differences exist in the drugs used, dosages, and treatment cycles across different studies, making it difficult to quantify intervention intensity and control intervention consistency, which impacts the rigor of comparisons. Most studies have short follow-up periods, and core issues such as long-term efficacy, recurrence control ability, and reproductive outcomes lack sufficient evidence to support, making it challenging to comprehensively evaluate the lasting value of integrated traditional Chinese and biomedicine regimens. In addition, the lack of direct comparisons between some treatment measures has led to incomplete connections in the network structure, limiting the interpretive power of SUCRA rankings and indirect comparisons.

Future research should focus on standardizing TCM syndrome classification and improving reporting norms, while strengthening the structural integrity of RCT study designs and ensuring continuous follow-up. Meanwhile, strategies such as “dosage quantification” and “process evaluation” for TCM interventions can be introduced to enhance the reproducibility and evaluability of TCM treatments. Based on in-depth mechanism exploration, more attention should be paid to outcome indicators with greater clinical translational value, such as fertility prognosis and long-term recurrence. These efforts aim to provide higher-quality evidence-based support for integrated traditional Chinese and biomedicine in the treatment of endometriosis.

## Data Availability

The raw data supporting the conclusions of this article will be made available by the authors, without undue reservation.

## Footnotes

1
http://mpns.kew.org/mpns-portal/

2
http://www.plantsoftheworldonline.org
