# Supplementary material for: Blood-invigorating Chinese herbal medicines plus conventional therapy for endometriosis: a network meta-analysis of 107 randomized controlled trials
Source: Front Med (Lausanne). 2026 May 13;13:1776698. doi: 10.3389/fmed.2026.1776698 (PMC13212517; doi:10.3389/fmed.2026.1776698)
Supplement: Supplementary file 1 [file Table_1.pdf]

Supplementary Table 1 Composition and Taxonomic Validation of Each Formula

Formula Name	Composition (with Taxonomic Validation)	Pharmacopoeia Reference
Bushen Huoxue Sanyu Decoction	1. Rehmannia glutinosa (Gaertn.) DC. [Orobanchaceae; Rehmanniae radix praeparata]<br>2. Cornus officinalis Sieb. et Zucc. [Cornaceae; Corni fructus]<br>3. Angelica sinensis (Oliv.) Diels [Apiaceae; Angelicae sinensis radix]<br>4. Ligusticum chuanxiong Hort. [Apiaceae; Ligustici chuanxiong rhizoma]<br>5. Salvia miltiorrhiza Bunge [Lamiaceae; Salviae miltiorrhizae radix et rhizoma]<br>6. Corydalis yanhusuo (Y.H.Chou & Chun C.Hsu) W.T.Wang ex Z.Y.Su & C.Y.Wu [Papaveraceae; Corydalis rhizoma]<br>7. Curcuma zedoaria (Christm.) Roscoe [Zingiberaceae; Curcumae rhizoma]<br>8. Panax notoginseng (Burkill) F.H.Chen ex C.H.Chow [Araliaceae; Notoginseng radix et rhizoma]	Chinese Pharmacopoeia (2020 Edition)
Dahuang Zhechong Capsule	1. Rheum palmatum L. [Polygonaceae; Rhei radix et rhizoma]<br>2. Eupolyphaga sinensis Walker [Blattidae; Eupolyphagae seu Steleophragmatis eupolyphaga]<br>3. Persica vulgaris Mill. [Rosaceae; Persicae semen]<br>4. Prunus armeniaca L. [Rosaceae; Armeniacae semen]<br>5. Angelica sinensis (Oliv.) Diels [Apiaceae; Angelicae sinensis radix]<br>6. Paeonia lactiflora Pall. [Paeoniaceae; Paeoniae radix alba]<br>7. Ligusticum chuanxiong Hort. [Apiaceae; Ligustici chuanxiong rhizoma]<br>8. Scrophularia ningpoensis Hemsl. [Scrophulariaceae; Scrophulariae radix]<br>9. Glycyrrhiza uralensis Fisch. [Fabaceae; Glycyrrhizae radix et rhizoma]	Chinese Pharmacopoeia (2020 Edition)
Dingkun Pill	1. Ginseng panax C.A.Mey. [Araliaceae; Ginseng radix et rhizoma]<br>2. Astragalus membranaceus (Fisch.) Bunge [Fabaceae; Astragali radix]<br>3. Angelica sinensis (Oliv.) Diels [Apiaceae; Angelicae sinensis radix]<br>4. Paeonia lactiflora Pall. [Paeoniaceae; Paeoniae radix alba]<br>5. Ligusticum chuanxiong Hort. [Apiaceae; Ligustici chuanxiong rhizoma]<br>6. Rehmannia glutinosa (Gaertn.) DC. [Orobanchaceae; Rehmanniae radix praeparata]<br>7. Cornus officinalis Sieb. et Zucc. [Cornaceae; Corni fructus]<br>8. Cyperus rotundus L. [Cyperaceae; Cyperi rhizoma]<br>9. Corydalis yanhusuo (Y.H.Chou & Chun C.Hsu) W.T.Wang ex Z.Y.Su & C.Y.Wu [Papaveraceae; Corydalis rhizoma]	Chinese Pharmacopoeia (2020 Edition)

	10. Poria cocos (Schw.) Wolf [Polyporaceae; Poria]<br>11. Glycyrrhiza uralensis Fisch. [Fabaceae; Glycyrrhizae radix et rhizoma]	
Danggui Shaoyao Powder	1. Angelica sinensis (Oliv.) Diels [Apiaceae; Angelicae sinensis radix]<br>2. Paeonia lactiflora Pall. [Paeoniaceae; Paeoniae radix alba]<br>3. Ligusticum chuanxiong Hort. [Apiaceae; Ligustici chuanxiong rhizoma]<br>4. Poria cocos (Schw.) Wolf [Polyporaceae; Poria]<br>5. Alisma orientale (Sam.) Juzep. [Alismataceae; Alismatis rhizoma]<br>6. Atractylodes macrocephala Koidz. [Asteraceae; Atractylodis macrocephalae rhizoma]	Chinese Pharmacopoeia (2020 Edition)
Eleng Capsule	1. Curcuma zedoaria (Christm.) Roscoe [Zingiberaceae; Curcumae rhizoma]<br>2. Sparganium stoloniferum (Graebn.) Buch.-Ham. ex Juz. [Typhaceae; Sparganii rhizoma]<br>3. Salvia miltiorrhiza Bunge [Lamiaceae; Salviae miltiorrhizae radix et rhizoma]<br>4. Angelica sinensis (Oliv.) Diels [Apiaceae; Angelicae sinensis radix]<br>5. Ligusticum chuanxiong Hort. [Apiaceae; Ligustici chuanxiong rhizoma]<br>6. Cyperus rotundus L. [Cyperaceae; Cyperi rhizoma]	Chinese Pharmacopoeia (2020 Edition)
Gongliuxiao (Preparation)	1. Astragalus membranaceus (Fisch.) Bunge [Fabaceae; Astragali radix]<br>2. Codonopsis pilosula (Franch.) Nannf. [Campanulaceae; Codonopsis radix]<br>3. Angelica sinensis (Oliv.) Diels [Apiaceae; Angelicae sinensis radix]<br>4. Ligusticum chuanxiong Hort. [Apiaceae; Ligustici chuanxiong rhizoma]<br>5. Salvia miltiorrhiza Bunge [Lamiaceae; Salviae miltiorrhizae radix et rhizoma]<br>6. Curcuma zedoaria (Christm.) Roscoe [Zingiberaceae; Curcumae rhizoma]<br>7. Sparganium stoloniferum (Graebn.) Buch.-Ham. ex Juz. [Typhaceae; Sparganii rhizoma]<br>8. Hedyotis diffusa Willd. [Rubiaceae; Hedyotidis diffusae herba]<br>9. Scutellaria baicalensis Georgi [Lamiaceae; Scutellariae radix]<br>10. Prunus persica (L.) Batsch [Rosaceae; Persicae semen]	Chinese Pharmacopoeia (2020 Edition)
Guizhi Fuling Pill/Capsule	1. Cinnamon cassia Presl [Lauraceae; Cinnamomi ramulus]<br>2. Poria cocos (Schw.) Wolf [Polyporaceae; Poria]<br>3. Paeonia suffruticosa Andr. [Paeoniaceae; Moutan cortex]<br>4. Paeonia lactiflora Pall. [Paeoniaceae; Paeoniae radix alba]<br>5. Persica vulgaris Mill. [Rosaceae; Persicae semen]	Chinese Pharmacopoeia (2020 Edition)

Guizhi Fuling Decoction	1. Cinnamon cassia Presl [Lauraceae; Cinnamomi ramulus]<br>2. Poria cocos (Schw.) Wolf [Polyporaceae; Poria]<br>3. Paeonia suffruticosa Andr. [Paeoniaceae; Moutan cortex]<br>4. Paeonia lactiflora Pall. [Paeoniaceae; Paeoniae radix alba]<br>5. Persica vulgaris Mill. [Rosaceae; Persicae semen]<br>6. Glycyrrhiza uralensis Fisch. [Fabaceae; Glycyrrhizae radix et rhizoma]	Chinese Pharmacopoeia (2020 Edition)
Hongjin Xiaojie (Preparation)	1. Rhizoma sparganii stoloniferi [Typhaceae; Sparganii rhizoma]<br>2. Curcuma zedoaria (Christm.) Roscoe [Zingiberaceae; Curcumae rhizoma]<br>3. Fritillaria thunbergii Miq. [Liliaceae; Fritillariae thunbergii bulbus]<br>4. Citrus reticulata Blanco [Rutaceae; Citri reticulatae pericarpium]<br>5. Cyperus rotundus L. [Cyperaceae; Cyperi rhizoma]<br>6. Angelica sinensis (Oliv.) Diels [Apiaceae; Angelicae sinensis radix]<br>7. Salvia miltiorrhiza Bunge [Lamiaceae; Salviae miltiorrhizae radix et rhizoma]<br>8. Glycyrrhiza uralensis Fisch. [Fabaceae; Glycyrrhizae radix et rhizoma]	Chinese Pharmacopoeia (2020 Edition)
Hongteng Formula	1. Sargentodoxa cuneata (Oliv.) Rehd. et Wils. [Lardizabalaceae; Sargentodoxae caulis]<br>2. Patrinia scabiosaefolia Fisch. ex Trev. [Caprifoliaceae; Patriniae scabiosifoliae radix]<br>3. Corydalis yanhusuo (Y.H.Chou & Chun C.Hsu) W.T.Wang ex Z.Y.Su & C.Y.Wu [Papaveraceae; Corydalis rhizoma]<br>4. Cyperus rotundus L. [Cyperaceae; Cyperi rhizoma]<br>5. Taraxacum mongolicum Hand.-Mazz. [Asteraceae; Taraxaci herba]<br>6. Scutellaria baicalensis Georgi [Lamiaceae; Scutellariae radix]	Chinese Pharmacopoeia (2020 Edition)
Jingtong Yushu Granule	1. Angelica sinensis (Oliv.) Diels [Apiaceae; Angelicae sinensis radix]<br>2. Ligusticum chuanxiong Hort. [Apiaceae; Ligustici chuanxiong rhizoma]<br>3. Paeonia lactiflora Pall. [Paeoniaceae; Paeoniae radix alba]<br>4. Cyperus rotundus L. [Cyperaceae; Cyperi rhizoma]<br>5. Corydalis yanhusuo (Y.H.Chou & Chun C.Hsu) W.T.Wang ex Z.Y.Su & C.Y.Wu [Papaveraceae; Corydalis rhizoma]<br>6. Glycyrrhiza uralensis Fisch. [Fabaceae; Glycyrrhizae radix et rhizoma]	Chinese Pharmacopoeia (2020 Edition)
Shaofu Zhuyu Decoction	1. Foeniculum vulgare Mill. [Apiaceae; Foeniculi fructus]<br>2. Lindera aggregata (Sims) Kosterm. [Lauraceae; Linderae radix]	Chinese Pharmacopoeia (2020 Edition)

	3. Corydalis yanhusuo (Y.H.Chou & Chun C.Hsu) W.T.Wang ex Z.Y.Su & C.Y.Wu [Papaveraceae; Corydalis rhizoma]<br>4. Cyperus rotundus L. [Cyperaceae; Cyperi rhizoma]<br>5. Angelica sinensis (Oliv.) Diels [Apiaceae; Angelicae sinensis radix]<br>6. Ligusticum chuanxiong Hort. [Apiaceae; Ligustici chuanxiong rhizoma]<br>7. Zingiber officinale Rosc. [Zingiberaceae; Zingiberis rhizoma recens]<br>8. Allium macrostemon Bunge [Amaryllidaceae; Allii macrostemonis bulbus]<br>9. Prunus persica (L.) Batsch [Rosaceae; Persicae semen]<br>10. Prunus armeniaca L. [Rosaceae; Armeniacae semen]	
Shaofu Zhuyu Granule	1. Foeniculum vulgare Mill. [Apiaceae; Foeniculi fructus]<br>2. Lindera aggregata (Sims) Kosterm. [Lauraceae; Linderae radix]<br>3. Corydalis yanhusuo (Y.H.Chou & Chun C.Hsu) W.T.Wang ex Z.Y.Su & C.Y.Wu [Papaveraceae; Corydalis rhizoma]<br>4. Cyperus rotundus L. [Cyperaceae; Cyperi rhizoma]<br>5. Angelica sinensis (Oliv.) Diels [Apiaceae; Angelicae sinensis radix]<br>6. Ligusticum chuanxiong Hort. [Apiaceae; Ligustici chuanxiong rhizoma]<br>7. Zingiber officinale Rosc. [Zingiberaceae; Zingiberis rhizoma recens]<br>8. Allium macrostemon Bunge [Amaryllidaceae; Allii macrostemonis bulbus]<br>9. Prunus persica (L.) Batsch [Rosaceae; Persicae semen]<br>10. Prunus armeniaca L. [Rosaceae; Armeniacae semen]	Chinese Pharmacopoeia (2020 Edition)
Sanjie Zhentong Capsule	1. Rhizoma sparganii stoloniferi [Typhaceae; Sparganii rhizoma]<br>2. Curcuma zedoaria (Christm.) Roscoe [Zingiberaceae; Curcumae rhizoma]<br>3. Corydalis yanhusuo (Y.H.Chou & Chun C.Hsu) W.T.Wang ex Z.Y.Su & C.Y.Wu [Papaveraceae; Corydalis rhizoma]<br>4. Panax notoginseng (Burkill) F.H.Chen ex C.H.Chow [Araliaceae; Notoginseng radix et rhizoma]	Chinese Pharmacopoeia (2020 Edition)
Tongjingling Granule	1. Angelica sinensis (Oliv.) Diels [Apiaceae; Angelicae sinensis radix]<br>2. Ligusticum chuanxiong Hort. [Apiaceae; Ligustici chuanxiong rhizoma]<br>3. Paeonia lactiflora Pall. [Paeoniaceae; Paeoniae radix alba]<br>4. Cyperus rotundus L. [Cyperaceae; Cyperi rhizoma]<br>5. Corydalis yanhusuo (Y.H.Chou & Chun C.Hsu) W.T.Wang ex Z.Y.Su & C.Y.Wu [Papaveraceae; Corydalis rhizoma]	Chinese Pharmacopoeia (2020 Edition)

	6. Zingiber officinale Rosc. [Zingiberaceae; Zingiberis rhizoma recens]<br>7. Glycyrrhiza uralensis Fisch. [Fabaceae; Glycyrrhizae radix et rhizoma]	
Xuefu Zhuyu Decoction	1. Angelica sinensis (Oliv.) Diels [Apiaceae; Angelicae sinensis radix]<br>2. Ligusticum chuanxiong Hort. [Apiaceae; Ligustici chuanxiong rhizoma]<br>3. Paeonia lactiflora Pall. [Paeoniaceae; Paeoniae radix rubra]<br>4. Persica vulgaris Mill. [Rosaceae; Persicae semen]<br>5. Carthamus tinctorius L. [Asteraceae; Carthami flos]<br>6. Platycodon grandiflorus (Jacq.) A.DC. [Campanulaceae; Platycodonis radix]<br>7. Bupleurum chinense DC. [Apiaceae; Bupleuri radix]<br>8. Citrus aurantium L. [Rutaceae; Aurantii fructus]<br>9. Rehmannia glutinosa (Gaertn.) DC. [Orobanchaceae; Rehmanniae radix]<br>10. Glycyrrhiza uralensis Fisch. [Fabaceae; Glycyrrhizae radix et rhizoma]	Chinese Pharmacopoeia (2020 Edition)
Xiaojin Capsule/ Tablet	1. Moschus berezovskii Flerov [Cervidae; Moschi moschiferi moschus]<br>2. Panax notoginseng (Burkill) F.H.Chen ex C.H.Chow [Araliaceae; Notoginseng radix et rhizoma]<br>3. Scolopendra subspinipes mutilans L. Koch [Scolopendridae; Scolopendrae scolopendra]<br>4. Eupolyphaga sinensis Walker [Blattidae; Eupolyphagae seu Steleophragmatis eupolyphaga]<br>5. Olibanum balsamum (L.) Merr. [Burseraceae; Olibani resinum]<br>6. Myrrha commiphora Engler [Burseraceae; Myrrhae resinum]<br>7. Rhizoma sparganii stoloniferi [Typhaceae; Sparganii rhizoma]<br>8. Curcuma zedoaria (Christm.) Roscoe [Zingiberaceae; Curcumae rhizoma]	Chinese Pharmacopoeia (2020 Edition)
Xiaoyi Decoction	1. Salvia miltiorrhiza Bunge [Lamiaceae; Salviae miltiorrhizae radix et rhizoma]<br>2. Curcuma zedoaria (Christm.) Roscoe [Zingiberaceae; Curcumae rhizoma]<br>3. Sparganium stoloniferum (Graebn.) Buch.-Ham. ex Juz. [Typhaceae; Sparganii rhizoma]<br>4. Persica vulgaris Mill. [Rosaceae; Persicae semen]<br>5. Carthamus tinctorius L. [Asteraceae; Carthami flos]<br>6. Angelica sinensis (Oliv.) Diels [Apiaceae; Angelicae sinensis radix]<br>7. Ligusticum chuanxiong Hort. [Apiaceae; Ligustici chuanxiong rhizoma]<br>8. Poria cocos (Schw.) Wolf [Polyporaceae; Poria]	Chinese Pharmacopoeia (2020 Edition)

Xiaozheng Decoction	1. Rhizoma sparganii stoloniferi [Typhaceae; Sparganii rhizoma]<br>2. Curcuma zedoaria (Christm.) Roscoe [Zingiberaceae; Curcumae rhizoma]<br>3. Salvia miltiorrhiza Bunge [Lamiaceae; Salviae miltiorrhizae radix et rhizoma]<br>4. Persica vulgaris Mill. [Rosaceae; Persicae semen]<br>5. Carthamus tinctorius L. [Asteraceae; Carthami flos]<br>6. Angelica sinensis (Oliv.) Diels [Apiaceae; Angelicae sinensis radix]<br>7. Paeonia lactiflora Pall. [Paeoniaceae; Paeoniae radix rubra]<br>8. Cyperus rotundus L. [Cyperaceae; Cyperi rhizoma]	Chinese Pharmacopoeia (2020 Edition)
Xiaozheng Zhitong Decoction	1. Curcuma zedoaria (Christm.) Roscoe [Zingiberaceae; Curcumae rhizoma]<br>2. Sparganium stoloniferum (Graebn.) Buch.-Ham. ex Juz. [Typhaceae; Sparganii rhizoma]<br>3. Corydalis yanhusuo (Y.H.Chou & Chun C.Hsu) W.T.Wang ex Z.Y.Su & C.Y.Wu [Papaveraceae; Corydalis rhizoma]<br>4. Salvia miltiorrhiza Bunge [Lamiaceae; Salviae miltiorrhizae radix et rhizoma]<br>5. Angelica sinensis (Oliv.) Diels [Apiaceae; Angelicae sinensis radix]<br>6. Ligusticum chuanxiong Hort. [Apiaceae; Ligustici chuanxiong rhizoma]<br>7. Paeonia lactiflora Pall. [Paeoniaceae; Paeoniae radix rubra]<br>8. Glycyrrhiza uralensis Fisch. [Fabaceae; Glycyrrhizae radix et rhizoma]	Chinese Pharmacopoeia (2020 Edition)

Note:

All botanical drugs have been taxonomically validated via MPNS (http://mpns.kew.org/mpns-portal/) and Plants of the World Online (http://www.plantsoftheworldonline.org), following the unified format: "Scientific name (Author) [Family; Pharmacopoeial drug name]".

Animal medicinal materials (e.g., Moschus, Scolopendra) are labeled with their source families and medicinal parts in accordance with the Chinese Pharmacopoeia (2020 Edition).

The composition of each formula is based on reports from original studies and records in the Chinese Pharmacopoeia, ensuring clarity and consistency with clinical applications.

For formulas with the same name but different dosage forms (e.g., Pill/Capsule, Decoction/Granule),

the core components remain consistent, with differences only in excipients or preparation processes. Thus, the core composition is listed uniformly
